# Effect of inflammatory cytokines and plasma metabolome on OSA: a bidirectional two- sample Mendelian randomization study and mediation analysis

**DOI:** 10.3389/fimmu.2024.1416870

**Published:** 2024-09-16

**Authors:** Xin Sun, Congying Wang, Yuheng He, Kun Chen, YingZhang Miao

**Affiliations:** ^1^ Hebei General Hospital, Shijiazhuang, Hebei, China; ^2^ Hebei North University, Zhangjiakou, Hebei, China

**Keywords:** obstructive sleep apnea, 91 plasma proteins, plasma-based metabolites, bidirectional two-sample Mendelian randomization, mediation analysis

## Abstract

**Background:**

Obstructive sleep apnea (OSA) is a common sleep disorder. Inflammatory factors and plasma metabolites are important in assessing its progression. However, the causal relationship between them and OSA remains unclear, hampering early clinical diagnosis and treatment decisions.

**Methods:**

We conducted a large-scale study using data from the FinnGen database, with 43,901 cases and 366,484 controls for our discovery MR analysis. We employed 91 plasma proteins from 11 cohorts (totaling 14,824 participants of European descent) as instrumental variables (IVs). Additionally, we conducted a GWAS involving 13,818 cases and 463,035 controls to replicate the MR analysis. We primarily used the IVW method, supplemented by MR Egger, weighted median, simple mode, and weighted mode methods. Meta-analysis was used to synthesize MR findings, followed by tests for heterogeneity, pleiotropy, and sensitivity analysis (LOO). Reverse MR analysis was also performed to explore causal relationships.

**Results:**

The meta-analysis showed a correlation between elevated Eotaxin levels and an increased risk of OSA (OR=1.050, 95% CI: 1.008-1.096; p < 0.05). Furthermore, we found that the increased risk of OSA could be attributed to reduced levels of X-11849 and X-24978 (decreases of 7.1% and 8.4%, respectively). Sensitivity analysis results supported the reliability of these findings.

**Conclusions:**

In this study, we uncovered a novel biomarker and identified two previously unknown metabolites strongly linked to OSA. These findings underscore the potential significance of inflammatory factors and metabolites in the genetic underpinnings of OSA development and prognosis.

## 1 Introduction

Obstructive sleep apnea (OSA) is a clinically common sleep breathing disorder characterized by recurrent upper airway collapse or obstruction during sleep, resulting in intermittent hypoxia, sleep fragmentation, and excessive daytime sleepiness (1). Presently, approximately 1 billion adults aged 30 to 69 are affected, with some countries experiencing a prevalence exceeding 50% (2). However, OSA remains significantly underestimated, particularly among women (3). OSA has been proved to be a risk factor for many diseases such as cardiovascular (4) and cerebrovascular diseases (5), and it can endanger life in serious cases. Continuous positive airway pressure therapy serves as a fundamental treatment for OSA, effectively enhancing neurocognitive function and reducing cardiovascular and cerebrovascular complications (6). Nonetheless, OSA progresses slowly, often leading patients to seek medical attention only when complications arise (7). Consequently, there’s a pressing need for more effective diagnostic and treatment strategies to mitigate adverse health outcomes.

Inflammation serves as a natural response to bodily damage, aiding in the reduction and repair of injuries. However, prolonged and excessive inflammation can lead to immune system dysregulation, macromolecular damage, metabolic alterations, and other adverse effects (8). In patients with obstructive sleep apnea (OSA), the repeated cycles of hypoxia and reoxygenation result in chronic intermittent hypoxia, leading to increased systemic oxidative stress. Additionally, inflammatory factors from the local upper airway mucosa enter the systemic bloodstream, contributing to systemic chronic inflammation (9). Previous research has focused on evaluating the impact of inflammatory markers such as CRP, IL-6, IL-8, and TNF-α on OSA severity, highlighting a strong association between OSA and systemic inflammation (10, 11). Possible pathogenesis may be obesity, pro-inflammatory diet, and lack of exercise. Inflammation can make OSA susceptible by weakening upper airway muscles, altering muscle denervation, reducing muscle contractility, and specifically increasing upper airway wettability during sleep (12–14).

Repeated exposure to hypoxic conditions alters gene transcription and post-translational protein modifications, leading to changes in plasma concentrations of lipids, proteins, and other biological compounds (15). With the availability of large metabolomic datasets, metabolites are increasingly used as biomarkers for disease progression. Previous studies have demonstrated that metabolites serve as functional intermediates, aiding in understanding the relationship between genetic variation and metabolites to elucidate disease mechanisms (16, 17). Research indicates that the pathogenesis of cardiovascular disease and metabolic complications associated with OSA may be significantly linked to specific metabolic changes. Mendelian randomization studies have identified associations between OSA and more than ten metabolites, including the plasma metabolite 3-Dehydrocarnitine, including plasma metabolite 3-Dehydrocarnitine, and OSA. The biosynthetic pathway of valine, leucine, and isoleucine is implicated in OSA pathogenesis (18). However, there is a lack of exploration into how inflammation alters metabolic pathways in the development of OSA and its complications. Therefore, understanding the involvement of metabolites in inflammation is critical for unraveling OSA pathogenesis and identifying potential therapeutic targets.

Mendelian randomization (MR) has emerged as a powerful method for establishing causal relationships in genetic epidemiology, circumventing the limitations of traditional observational studies. While observational studies may struggle to eliminate environmental confounders and establish definitive causal links, MR offers a widely accepted approach for addressing these challenges. By leveraging genetic variants as instrumental variables, MR minimizes the impact of confounding factors and reverse causation, facilitating more accurate causal inference between exposure and outcome. While randomized controlled trials (RCTs) remain the gold standard for confirming causal associations, their practical implementation is often constrained by ethical, temporal, and spatial considerations. In this study, MR was employed to explore the relationship between 91 plasma inflammatory proteins and obstructive sleep apnea (OSA). The primary aim was to elucidate the causal connections between inflammatory proteins and OSA, shedding light on the underlying mechanisms contributing to its development. By utilizing MR, this research endeavors to provide valuable insights into the etiology of OSA and potentially inform future therapeutic interventions.

## 2 Methods

### 2.1 Study design

**Figure 1:**
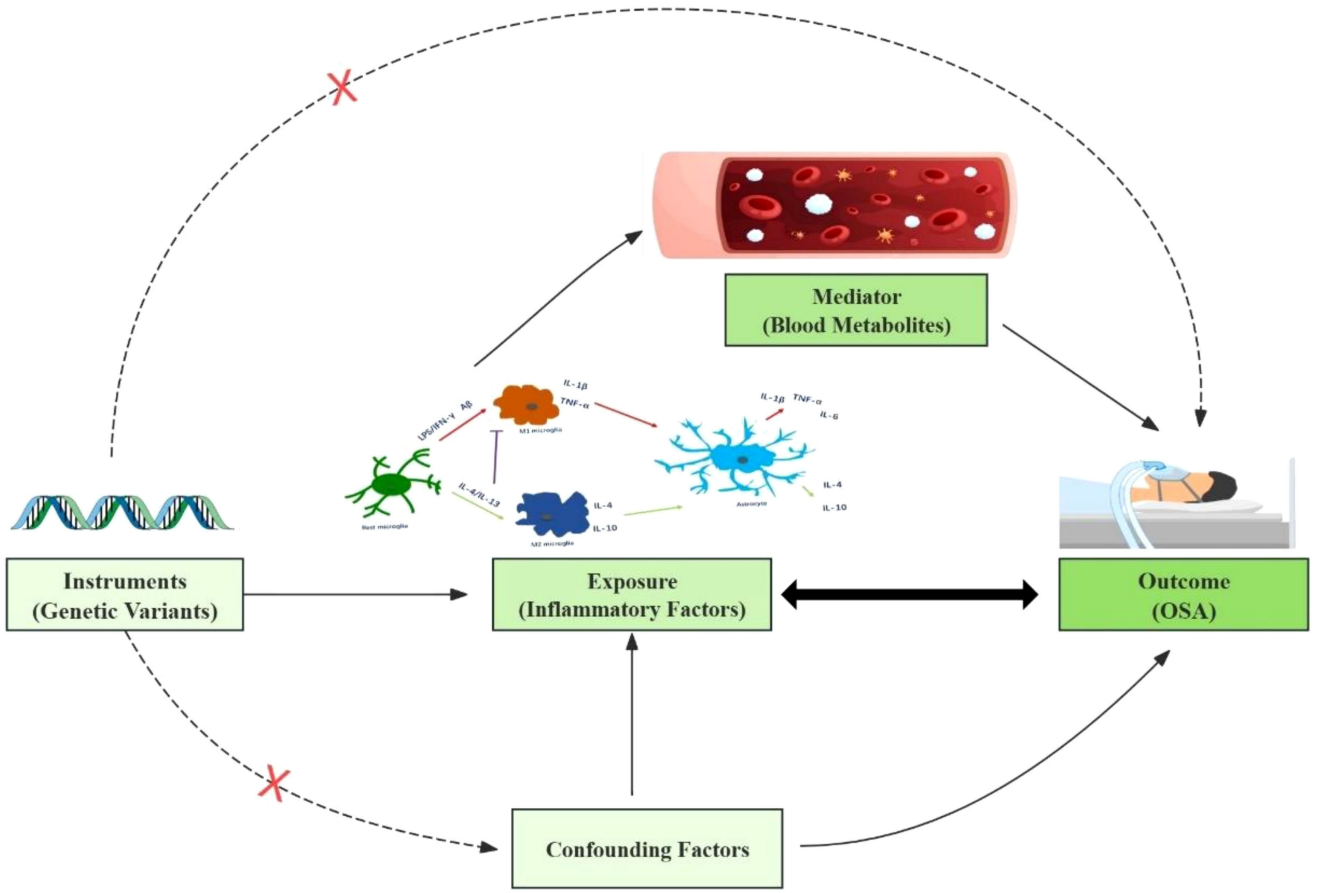
This study design is based on three MR assumptions: IVs must be closely linked to the exposure, affect the outcome solely through the exposure, and have no association with confounders.

A comprehensive investigation employing an integrated genetic approach was designed (**Figure 1**). The causal interpretation of MR estimates relies on three assumptions: IVs must, first and foremost, be closely linked to the exposure factor(s). Secondly, IVs only associate with the outcome via the exposures. Third, no possible confounding factor was associated with IVs. The STORBE-MR (Strengthening the Reporting of Observational Studies in Epidemiology using Mendelian Randomization) checklist was completed for this observational study.

### 2.2 Data sources

**Figure 2:**
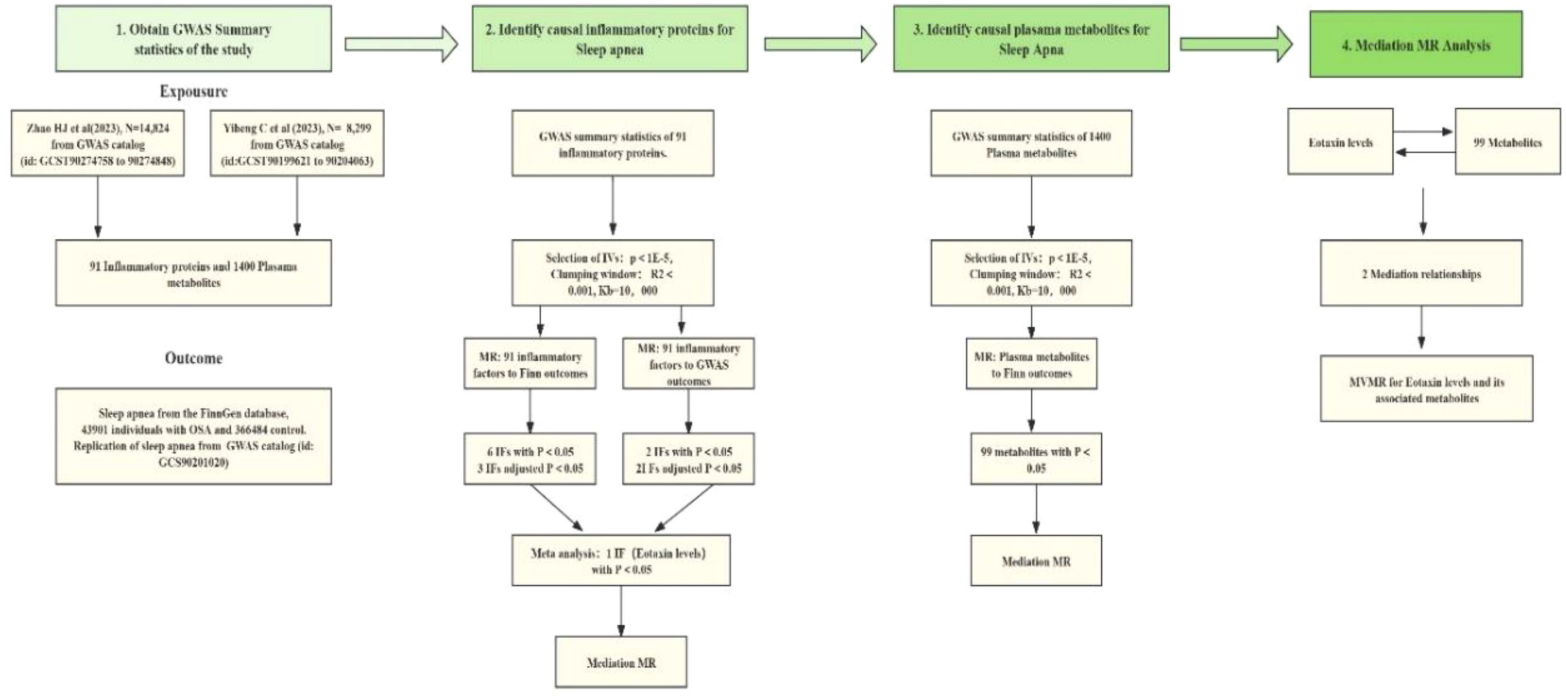
The bidirectional and mediation Mendelian Randomization (MR) analysis included these steps: First, a two-sample bidirectional MR study assessed the causal relationship between 91 inflammatory factors and OSA using data from the FinnGen and GWAS catalog databases. Sensitivity analyses and FDR correction were applied, including exposures with a P value below 0.05 after accounting for pleiotropy in the meta-analysis. Only robust factors in the meta-analysis proceeded to mediation analysis. Second, 1400 blood metabolites were selected for mediation analysis. Finally, a two-step MR analysis identified potential mediating metabolites by evaluating the effect of inflammatory factors on metabolites and metabolites on OSA.

For Mendelian randomization (MR), it is crucial that the genetic variants used are representative of cytokines. Therefore, we selected SNPs associated with 91 plasma inflammatory proteins. Summary statistics for these 91 plasma inflammatory proteins (19) were collected from the latest GWAS Catalog study (accession numbers GCST90274758 to GCST90274848). This comprehensive analysis involved 14,824 European participants across 11 cohorts, providing insights into various inflammation-related cytokines (**Figure 2**). The number of SNPs used as IVs ranged from 1 to 623(median, 33).

Additionally, summary statistics of plasma-based metabolites (20) were acquired from the most recent GWAS Catalog database under the study accession numbers GCST90199621-GCS90201020. This study encompassed 1,091 plasma metabolites and the ratios of 309 metabolites from 8,299 European individuals. Among these metabolites, 850 were known and could be categorized into 8 broad metabolic groups: lipid, amino acid, xenobiotics, nucleotides, cofactors and vitamins, carbohydrates, peptides, and energy. The remaining metabolites were partially characterized molecules and unknown.

Furthermore, GWAS summary data for OSA were extracted from the FinnGen database (21), which comprises information from 43,901 individuals with OSA and 366,484 control subjects. This GWAS data tested up to 21,306,327 SNPs related to OSA. The diagnosis of OSA (Obstructive Sleep Apnea) was confirmed using the International Classification Criteria for Sleep Disorders. This requires either the presence of signs or symptoms (e.g., sleepiness, fatigue, insomnia, snoring, subjective nocturnal respiratory disturbances, or observed apnea) or an associated medical or psychiatric disorder (e.g., hypertension, coronary artery disease, atrial fibrillation, congestive heart failure, stroke, diabetes, cognitive dysfunction, or mood disorder) along with five or more predominantly obstructive respiratory events per hour. Alternatively, a frequency of obstructive respiratory events of 15 per hour satisfies the criteria, even in the absence of associated symptoms or disorders.

FinnGen is a large-scale genomics initiative analyzing over 500,000 Finnish biobank samples, correlating genetic variation with health data to understand disease mechanisms and predispositions. It is a collaboration between research organizations, biobanks within Finland, and international industry partners. The diagnosis of OSA was made using the International Statistical Classification of Diseases and Related Health Problems codes, obtained from the Finnish National Hospital Discharge Register and the Cause of Death Register. Specifically, the codes used were ICD-10: G47.3 and ICD-9: 3472A.

Despite implementing strict criteria and conducting sensitivity analyses to ensure data reliability, the IVW analysis was repeated using a supplementary dataset of 476,853 individuals (13,818 SAS patients, 463,035 controls) obtained from the GWAS catalog (GCST90018916). This GWAS dataset analyzed up to 13,429,585 SNPs related to OSA. The diagnosis of OSA was based on the ICD-10 code G47.3 (22).

### 2.3 Instrumental variable selection

Given the limited number of SNPs identified through stringent filtering, we relaxed the significance threshold for the 91 plasma inflammatory proteins and plasma-based metabolites to P < 1 × 10^-5, without altering other selection criteria. Independent SNPs were then clumped to a linkage disequilibrium (LD) threshold of r^2 < 0.001 and kb = 10,000. Considering that the GWAS data for OSA have a sufficient number of significant SNPs, we aimed to ensure that the instrumental variables in the model have adequate strength to avoid potential weak instrument bias and violations of the exclusion restriction. Therefore, we chose a genome-wide significance threshold of P < 5 × 10^-8 as the criterion for including SNPs, with LD parameters set to r^2 < 0.001 and kb = 10,000 as conditions for removing LD. In cases where there were no common SNPs shared between the exposure and outcome variables, proxies from the 1000 Genomes European reference panel with a linkage disequilibrium coefficient (r^2) of at least 0.8 were incorporated.

Then, we calculated the R^2^ and F values for each SNP and excluded SNPs with an F value less than 10. Palindromic SNPs with intermediate-effect allele frequencies were excluded. Finally, the preserved SNPs were used for MR analysis.

### 2.4 Statistical analysis and sensitivity analysis

All MR analyses were performed using the “TwoSampleMR” and “MRPRESSO” software packages in R software (version 4.2.2), with inverse variance weighting (IVW) (23) as the primary method due to its precision and lack of bias. In addition to IVW, MR Egger (24), Weighted median (25), and Weighted mode analyses (26) were employed to mitigate potential confounders. False discovery rate (FDR) (27) corrected p-values were computed using the Benjamin-Hochberg method to address multiple testing. A post-FDR adjusted p-value below 0.05 indicated a relatively convincing causal relationship. However, MR analysis outcomes with an FDR greater than 0.05 but IVW method p-value less than 0.05 were considered to have a nominally significant causal relationship and were identified as potential risk factors.

Discovery and replication MR analyses findings were combined for a meta-analysis, integrating at least two reliably identified types. A merged p-value under 0.05 was considered significant, with interpretations based on odds ratios (ORs). To ensure result robustness, heterogeneity testing, level pleiotropy testing, and Leave-One-Out (LOO) sensitivity analysis were performed. Initially, MR-PRESSO (28) was utilized to detect horizontal pleiotropy. If detected, outliers were removed for subsequent MR reanalysis. If horizontal pleiotropy was absent, the Cochran Q test (29) was applied, with a Q value over 0.05 indicating no heterogeneity. LOO analysis evaluated each SNP’s impact and identified any outliers.

### 2.5 Genetic analyses to elucidate causality

Genetic correlation is a crucial population parameter that delineates the shared genetic architecture among complex traits and diseases. This correlation can significantly impact Mendelian Randomization (MR) estimates, potentially leading to confounding of causal effects. To mitigate this risk and ensure the robustness of causal inference, we employed linkage disequilibrium score regression (LDSC) (30). This method allowed us to meticulously examine the genetic correlation between the selected 91 plasma inflammatory proteins and OSA. By assessing the genetic overlap between these traits, we aimed to ascertain the independence of the genetic instruments used in MR analysis, thereby enhancing the reliability of causal effect estimates.

### 2.6 Analysis of confounding and reverse causation

Despite conducting an array of sensitivity analyses to assess the horizontal pleiotropy of the MR results and identify any SNPs that contravened the MR assumptions, it’s possible that some residual confounding SNPs may persist. To address this concern, we examined the SNPs in the instrumental variables (IVs) using the Phenoscanner V2 website (http://www.phenoscanner.medschl.cam.ac.uk/). This allowed us to evaluate the association of each SNP with known OSA risk factors, such as smoking, obesity, age, and others.

Furthermore, to explore whether OSA has any causal effect on the identified 91 plasma inflammatory proteins, we performed reverse MR analysis. In this analysis, OSA was considered as the exposure and the identified 91 plasma inflammatory proteins were treated as the outcome for MR analysis. This approach enabled us to investigate the potential causal relationship between OSA and the inflammatory proteins, using OSA-related SNPs as instrumental variables.

### 2.7 Mediation analyses

Mediation analysis (31) aims to assess the pathway from exposure to outcome through a mediator, thereby elucidating potential mechanisms underlying the effect of exposure on outcome. In this study, mediation analysis focused on inflammatory proteins, plasma metabolites, and OSA. Initially, the causal relationship between inflammatory proteins and OSA was evaluated using two-sample Mendelian randomization (MR) methods to obtain beta (A). Subsequently, multivariable Mendelian randomization (MVMR) was employed to identify plasma metabolites that still exhibited a causal relationship with OSA after adjusting for inflammatory proteins, yielding beta (B). This ensured that the mediating effects on outcomes remained independent of the exposure. The mediation effect was then calculated using a two-step MR approach: mediation effect = beta (A) × beta (B). The total effect of inflammatory proteins on OSA was determined in the preceding two-sample MR analysis, where the direct effect was computed as (total effect − mediation effect).

### 2.8 Ethical approval and consent to participate

Ethical clearance and consent for participation were obtained for each individual study included in this GWAS meta-analysis. Approval was granted by the appropriate Institutional Review Board, and either participants or their proxies, such as caregivers or legal guardians, provided informed consent prior to their involvement in the study. The data utilized in this research are publicly accessible.

## 3 Results

### 3.1 Effect of inflammatory proteins on OSA risk

Utilizing the IVW method as the primary evaluative approach, we identified 7 inflammatory proteins exhibiting a nominally significant causal association with OSA (P < 0.05) (**Table 1**). Following FDR correction, 5 inflammatory proteins were discovered to maintain a significant association (P < 0.05). The IVW reported that higher levels of Eotaxin levels (OR=1.051, 95% CI, 1.096-1.008; P = 0.043), CUB domain-containing protein 1 levels (OR=1.043, 95% CI, 1.084-1.005; P = 0.043), and Interleukin-20 receptor subunit alpha levels (OR=1.090, 95% CI, 1.150-1.003; P = 0.011) were associated with an elevated risk of OSA, while higher levels of Glial cell line-derived neurotrophic factor levels (OR=0.954, 95% CI, 0.998-0.911; P = 0.049) and Thymic stromal lymphopoietin levels (OR=0.950, 95% CI, 0.999-0.903; P = 0.047) associated with a decreased risk of the disease.

**Table 1:** Effects of inflammatory proteins on OSA risk (Finn outcome) in the study.

Inflammatory proteins	Methods	SNP(n)	OR (95%CI)	P	Adjust P	Heterogeneity		Pleiotropy	
						Q	P	intercept	P
Eotaxin levels	IVW	27	1.05(1.008-1.096)	0.018	0.037	31.33	0.21	0.005	0.23
	MR Egger	27	1.00(0.932-1.091)	0.833		29.56	0.24		
	WM	27	1.02(0.972-1.090)	0.318					
CD6 isoform levels	IVW	551	1.01(1.000-1.026)	0.039	0.054	554.81	0.43	0.0001	0.84
	MR Egger	551	1.01(0.989-1.034)	0.309		554.78	0.42		
	WM	551	0.99(0.969-1.028)	0.928					
CDCP1 levels	IVW	35	1.04(1.005-1.084)	0.025	0.043	52.43	0.02	-0.008	0.03
	MR Egger	35	1.10(1.040-1.182)	0.003		45.62	0.07		
	WM	35	1.07(1.023-1.129)	0.003					
GDNF levels	IVW	24	0.95(0.911-0.998)	0.042	0.049	28.67	0.19	-0.006	0.21
	MR Egger	24	0.99(0.920-1.073)	0.885		26.66	0.22		
	WM	24	0.96(0.911-1.031)	0.322					
Interleukin-20 levels	IVW	23	0.94(0.896-0.989)	0.017	0.059	16.00	0.81	-0.004	0.55
	MR Egger	23	0.96(0.870-1.080)	0.585		15.64	0.78		
	WM	23	0.95(0.893-1.016)	0.140					
IL-20RA levels	IVW	20	1.09(1.033-1.150)	0.001	0.011	20.82	0.34	0.006	0.39
	MR Egger	20	1.03(0.907-1.178)	0.619		19.99	0.33		
	WM	20	1.06(0.988-1.148)	0.097					
TSLP levels	IVW	22	0.95(0.903-0.999)	0.047	0.047	21.91	0.40	-0.0004	0.94
	MR Egger	22	0.95(0.846-1.074)	0.446		21.91	0.34		
	WM	22	0.94(0.881-1.014)	0.122					

CD6 isoform levels: T-cell surface glycoprotein CD6 isoform levels; CDCP1 levels: CUB domain-containing protein 1 levels; GDNF levels: Glial cell line-derived neurotrophic factor levels; IL-20RA levels: Interleukin-20 receptor subunit alpha levels; TSLP levels: Thymic stromal lymphopoietin levels; IVW, inverse variance weighted mode; OR, Odds ratio; 95%CI = 95% confidence interval.

**Figure 3:**
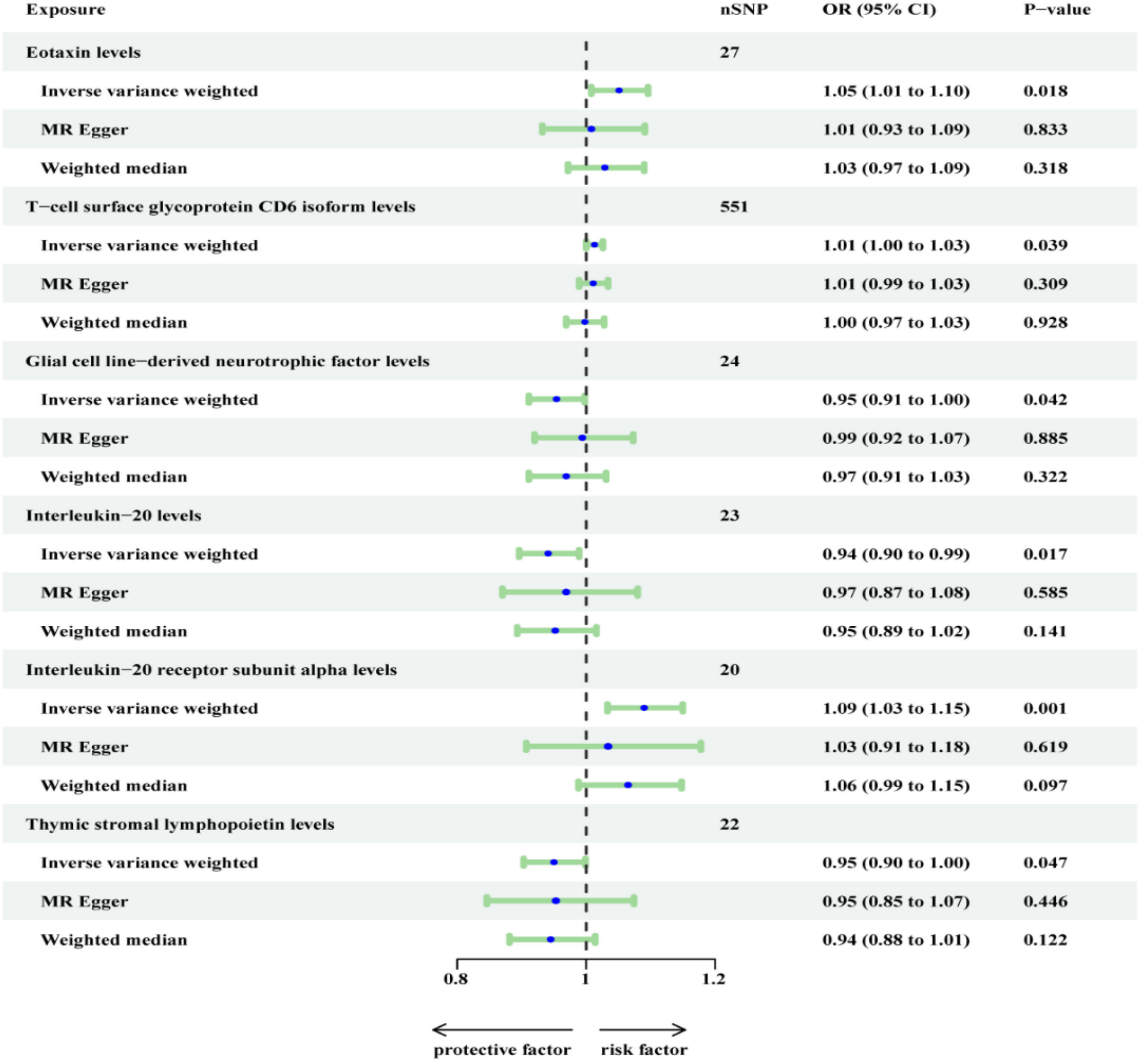
Re-analyzed the causal effects of inflammatory proteins on OSA (Finn outcome) after excluding CDCP1.

In the sensitivity analysis, CUB domain-containing protein 1 levels were found to exhibit horizontal pleiotropy. Consequently, we excluded it and conducted a re-analysis with FDR correction (**Figure 3**). However, it is regrettable to note that T-cell surface glycoprotein CD6 isoform levels (OR=1.013, 95% CI, 1.026-1.000; P = 0.058), Glial cell line-derived neurotrophic factor levels (OR=0.954, 95% CI, 0.998-0.911; P = 0.050), and Interleukin-20 levels (OR=0.941, 95% CI, 0.989-0.896; P = 0.051) did not maintain a significant association (**Supplementary Table 1**).

On the other hand, 3 inflammatory proteins were still found to maintain a significant association (P < 0.05). Specifically, Eotaxin levels (OR=1.051, 95% CI, 1.096-1.008; P = 0.037) and Interleukin-20 receptor subunit alpha levels (OR=1.090, 95% CI, 1.150-1.033; P = 0.009) were positively associated with OSA, while Thymic stromal lymphopoietin levels (OR=0.903, 95% CI, 0.999-0.903; P = 0.047) were negatively associated with OSA. In the supplementary table, these three inflammatory proteins from the MR-Egger and weighted median reported results in the same direction compared to IVW. The above results were visualized in scatter plots and forest plots, and SNP heterogeneity for the inflammatory proteins was satisfactory according to Cochran’s Q test, leave-one-SNP-out analysis, and funnel plots (**Supplementary Figures 1**-**24**).

### 3.2 Replication and meta-analysis

**Figure 4:**
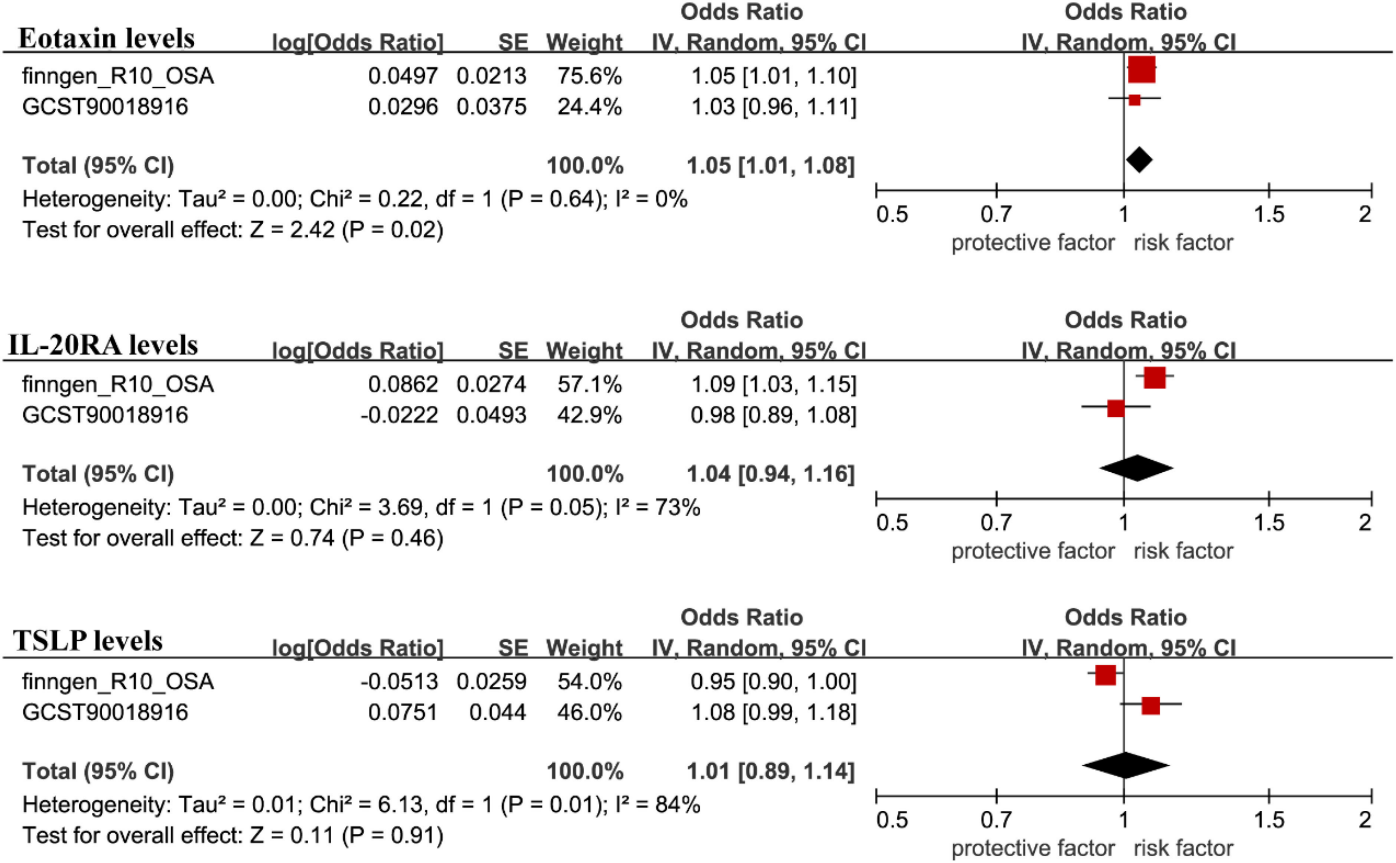
The GWAS catalog database was used for validation, and the filtered results were included in the meta-analysis with the FinnGen database findings.

We employed another cohort dataset for OSA obtained from the GWAS database and replicated the MR analysis described above. However, we found that the replication results for Interleukin-20 receptor subunit alpha levels showed considerable differences. Conversely, Eotaxin levels and Thymic stromal lymphopoietin levels showed similar results (**Supplementary Table 2**). To combine the findings, meta-analysis was conducted using random effects in R, which showed that Eotaxin levels yielded similar outcomes, while Thymic stromal lymphopoietin levels and Interleukin-20 receptor subunit alpha levels were excluded due to insignificant results (**Figure 4**).

### 3.3 Analysis of confounding and reverse causation

Then, we conducted a search for phenotypes associated with Eotaxin levels and found that, among the 28 SNPs related to Eotaxin levels, rs12075 was associated with obesity, while the other 27 SNPs were not associated with confounding factors (**Supplementary Table 3**). After excluding this SNP, the results for Eotaxin levels, remained significant (OR=1.059, 95% CI 1.016-1.104; P = 0.006). In the reverse MR analysis, Eotaxin levels did not show a reverse causal relationship with OSA. However, we found that Macrophage inflammatory protein 1a levels, C-C motif chemokine 28 levels, fms-related tyrosine kinase 3, Monocyte chemoattractant protein-3 levels, Adenosine deaminase levels, Fibroblast growth factor 19 levels, Monocyte chemoattractant protein-4 levels, Fractalkine levels, and STAM binding protein levels may have a reverse causal relationship with OSA (**Table 2**).

**Table 2:** Reverse MR analysis of OSA (FinnGen outcome) effects on inflammatory proteins.

exposure	outcome	IVW	OR (95%CI)	adjustP
Sleep apnea	Macrophage inflammatory protein 1a levels	0.005	1.19(1.051-1.348)	0.54
Sleep apnea	C-C motif chemokine 28 levels	0.019	0.86(0.768-0.977)	0.90
Sleep apnea	Fms-related tyrosine kinase 3	0.022	1.17(1.023-1.351)	0.68
Sleep apnea	Monocyte chemoattractant protein-3 levels	0.032	1.16(1.012-1.335)	0.73
Sleep apnea	Adenosine Deaminase levels	0.035	1.14(1.009-1.291)	0.64
Sleep apnea	Fibroblast growth factor 19 levels	0.038	0.87(0.772-0.992)	0.58
Sleep apnea	Monocyte chemoattractant protein-4	0.038	1.14(1.006-1.291)	0.51
Sleep apnea	Fractalkine levels	0.044	0.85(0.737-0.995)	0.50
Sleep apnea	STAM binding protein levels	0.044	1.13(1.003-1.285)	0.45

### 3.4 Analysis of genetic correlation

According to the results of the genetic correlation analysis, evidence of genetic correlation between Eotaxin levels (Rg = 0.041, Rg-se = 0.096, Rg-p = 0.665) in OSA is weak. This indicates that the shared genetic components did not confound the MR results.

### 3.5 Mediation analyses

**Figure 5:**
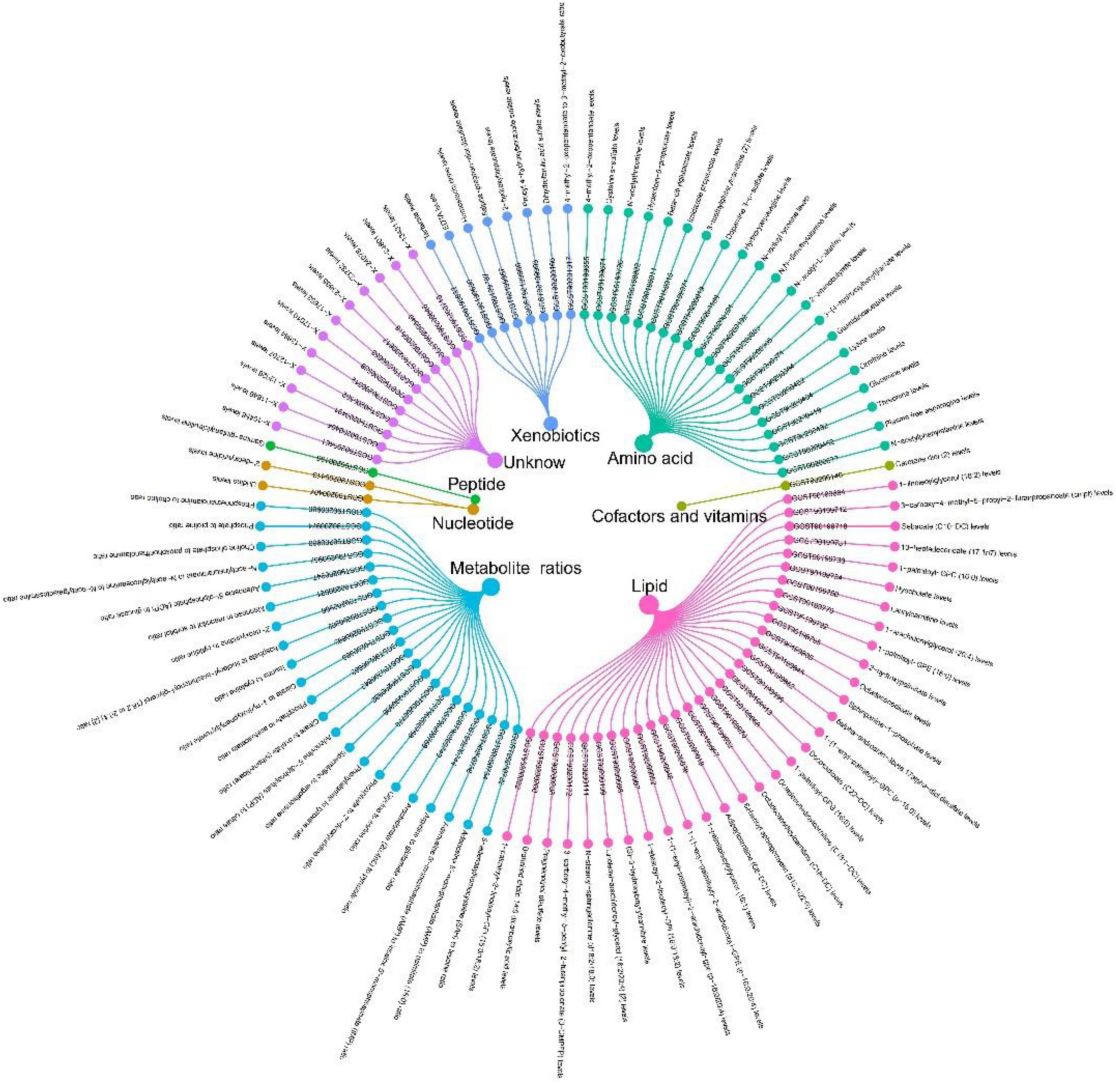
98 blood metabolites were found to be causally associated with OSA.

In the Two-sample Mendelian randomization (TSMR) analysis, we discovered that only 98 blood metabolites were causally associated with OSA (**Figure 5**). Further details on these 98 metabolites are provided in **Supplementary Table 4**. Among these 98 blood metabolites, Eotaxin levels were causally associated with only two: X-11849 levels (OR=0.881, 95% CI, 0.996-0.804; P = 0.007) and X-24978 levels (OR=1.119, 95% CI, 1.242-1.008; P = 0.034). These two unknown metabolites do not belong to any of the eight categories mentioned earlier. Subsequently, we conducted a Two-step Mediation MR to validate the mediating effects of these blood metabolites uncovered in TSMR. We calculated the indirect effect and proportion mediated by these metabolites. Overall, we observed indirect effects of X-11849 levels and X-24978 levels in the associations between Eotaxin levels and OSA, with a mediated proportion of 7.0% (P = 0.048) and 8.4% (P = 0.047), respectively.

## 4 Discussion

Our study represents the first to employ stringent Mendelian randomization methods, incorporating rigorous screening criteria. We utilized the most comprehensive inflammation-related GWAS data, encompassing up to 91 cytokines, along with the latest OSA cohort from the FinnGen database for MR analysis. Additionally, we supplemented this with the largest set of GWAS data for replication MR. Finally, through meta-analysis, we synthesized evidence revealing a causal relationship between elevated Eotaxin levels and an increased risk of OSA. Specifically, each additional standard deviation in Eotaxin levels was associated with a 5.2% increased risk of OSA.

This groundbreaking discovery represents a significant addition to existing literature. The newfound association underscores the need for further research to validate Eotaxin levels as a potential biomarker for both OSA prevention and treatment strategies. Moreover, our findings suggest that the heightened risk of OSA may be mediated by reduced levels of X-11849 and X-24978, with decreases of 7.1% and 8.4%, respectively.

Eotaxin levels play a crucial role in the immune system by binding to receptors, directing white blood cells, particularly eosinophils, to migrate and accumulate at sites of inflammation or infection. However, excessive expression of Eotaxin can lead to abnormal aggregation of white blood cells and inflammatory responses, worsening the symptoms and severity of the disease. Previous research has linked Eotaxin levels to asthma attacks (32–34), with evidence suggesting that Eotaxin can enhance the association between other chemokine genes on chromosome 17q21 and asthma, thereby exacerbating asthma symptoms and severity (35).

OSA and bronchial asthma share common etiology and influence each other, potentially due to increased Eotaxin levels in asthma patients (32, 33). Elevated plasma Eotaxin levels have also been observed in patients with chronic sinusitis and nasal polyps, likely due to repeated respiratory tract infections and allergic inflammation in the upper respiratory tract (34). This inflammation leads to nasal mucosa edema, thickening, and remodeling, resulting in upper airway stenosis and OSA. Furthermore, studies have found higher plasma Eotaxin levels in obese individuals, which decrease after exercise-induced weight loss (35, 36). Obesity, a known risk factor for OSA, likely influences OSA occurrence through this pathway.

Remarkably, a significant association between plasma Eotaxin levels and coronary atherosclerosis has been identified in patients with coronary atherosclerotic heart disease, along with a strong correlation with the degree of coronary artery disease (CAD) stenosis (37). Eotaxin not only promotes inflammation but also has a chemotactic effect on human microvascular endothelial cells, supporting vessel formation at sites of inflammation (38). This suggests that elevated plasma Eotaxin levels may serve as a novel marker for diffuse coronary atherosclerosis.

Recognizing the pivotal role metabolites may play in the interplay between inflammation and OSA, we conducted an MR analysis to explore potential associations. We identified 88 known metabolites and 11 unknown metabolites, including 23 metabolite ratios and 76 metabolites (64 known and 12 unknown) with potential causal links to OSA risk. Ultimately, only levels of X-11849 and X-24978 were implicated in the pathogenesis of OSA. Although the specific metabolites involved in mediation remain unknown, this finding may offer a target for future therapeutic endeavors.

Furthermore, this study investigated the impact of OSA on related inflammatory regulators. It was found that fibroblast growth factor 19 levels, known to have a protective effect on atherosclerosis in patients with type 2 diabetes, are down-regulated by OSA, thus weakening their protective effect and exacerbating outcomes, consistent with prior research (39, 40). Elevated levels of adenosine deaminase, fms-related tyrosine kinase 3 ligand, and fractalkine have been associated with autoimmune diseases, lymphoma, and atherosclerosis in animal and observational studies (41–47). However, findings on the effects of fractalkine inhibition on plaque formation have been contradictory (46, 47). Moreover, C-C motif chemokine 28 levels, highly expressed in malignant tumors such as pancreatic cancer and lymphoma, and upregulated levels of monocyte chemoattractant protein-3 and -4, are implicated in regulating inflammatory responses, particularly in allergic inflammation (48–52). Additionally, STAM binding protein levels play pivotal roles in cellular physiology and pathology, modulating signaling pathways, endocytosis, and protein conversion. Understanding their functions and molecular mechanisms may unveil potential therapeutic targets for various diseases (53).

In this study, MR analysis was utilized to identify inflammatory factors and metabolites potentially associated with OSA, incorporating bidirectional MR analysis and mediation analysis. Large GWAS data validation, meta-analysis integration, and stringent quality control measures were implemented to ensure reliable and stable detection results. The successful association of inflammatory factors with the plasma metabolome and the establishment of pathways linking inflammatory factors to OSA through plasma metabolites provide valuable insights into the metabolic mechanisms underlying OSA and its disease progression, holding significant clinical research value. Additionally, several inflammatory regulators were identified, such as monocyte chemoattractant protein-3 and -4, which may be implicated in allergic inflammation and autoimmune diseases, suggesting a potential mediating role of inflammatory factors between OSA and these conditions. Future studies should explore the mechanism of peripheral inflammation before and after OSA development at both basic and clinical levels, paving the way for new anti-inflammatory-based therapies to effectively prevent and improve its prognosis.

However, the study has some limitations. Due to the predominantly European dataset used, demographic stratification bias may have been introduced, limiting the generalizability of the conclusions to other racial groups. Therefore, further research involving diverse racial groups is warranted. Additionally, while several metabolites with a causal relationship with OSA were identified, there remain unproven metabolites whose role in the disease is not fully understood, hampering a comprehensive analysis and interpretation of the findings.

## 5 Conclusion

In this study, we discovered a novel biomarker and two unidentified metabolites that are strongly linked to OSA. This highlights the potential significance of inflammatory factors and metabolites in influencing the development and prognosis of OSA at a genetic level.

## Data Availability

The original contributions presented in the study are included in the article/**Supplementary Material**. Further inquiries can be directed to the corresponding author.
