# Supplementary material for: Effect of inflammatory cytokines and plasma metabolome on OSA: a bidirectional two- sample Mendelian randomization study and mediation analysis
Source: Front Immunol. 2024 Sep 16;15:1416870. doi: 10.3389/fimmu.2024.1416870 (PMC11439640; doi:10.3389/fimmu.2024.1416870)

Supplementary Table 1 excluded CDCP1 levels and conducted a re-analysis with FDR correction

Inflammatory proteins	Methods	SNP(n)	OR (95%CI)	P	Adjust P	Heterogeneity		Pleiotropy	
						Q	P	intercept	P
Eotaxin levels	IVW	27	1.05(1.008-1.096)	0.018	0.037	31.33	0.21	0.005	0.23
	MR Egger	27	1.00(0.932-1.091)	0.833		29.56	0.24		
	WM	27	1.02(0.972-1.090)	0.318					
CD6 isoform levels	IVW	551	1.01(1.000-1.026)	0.039	0.058	554.81	0.43	0.0001	0.84
	MR Egger	551	1.01(0.989-1.034)	0.309		554.78	0.42		
	WM	551	0.99(0.969-1.028)	0.928					
GDNF levels	IVW	24	0.95(0.911-0.998)	0.042	0.051	28.67	0.19	-0.006	0.21
	MR Egger	24	0.99(0.920-1.073)	0.885		26.66	0.22		
	WM	24	0.96(0.911-1.031)	0.322					
Interleukin-20 levels	IVW	23	0.94(0.896-0.989)	0.017	0.051	16.00	0.81	-0.004	0.55
	MR Egger	23	0.96(0.870-1.080)	0.585		15.64	0.78		
	WM	23	0.95(0.893-1.016)	0.140					
IL-20RA levels	IVW	20	1.09(1.033-1.150)	0.001	0.009	20.82	0.34	0.006	0.39
	MR Egger	20	1.03(0.907-1.178)	0.619		19.99	0.33		
	WM	20	1.06(0.988-1.148)	0.097					
TSLP levels	IVW	22	0.95(0.903-0.999)	0.047	0.047	21.91	0.40	-0.0004	0.94
	MR Egger	22	0.95(0.846-1.074)	0.446		21.91	0.34		
	WM	22	0.94(0.881-1.014)	0.122					

CD6 isoform levels: T-cell surface glycoprotein CD6 isoform levels; GDNF levels: Glial cell line-derived neurotrophic factor levels; IL-20RA levels: Interleukin-20 receptor subunit alpha levels; TSLP levels: Thymic stromal lymphopoietin levels

Supplementary Table 2 Replication and meta-analysis

Inflammatory proteins	Outcome	Methods	SNP(n)	OR (95%CI)	P	Weight	Overall effect	
							Z	P
Eotaxin levels	Finn	IVW	27	1.05(1.008-1.096)	0.018	75.07%	2.43	0.01
	Finn	MR Egger	27	1.00(0.932-1.091)	0.833			
	Finn	WM	27	1.02(0.972-1.090)	0.318			
	GWAS	IVW	29	1.03(0.957-1.108)	0.422	24.93%		
	GWAS	MR Egger	29	1.05(0.909-1.214)	0.504			
	GWAS	WM	29	1.09(0.983-1.210)	0.099			
IL-20RA levels	Finn	IVW	20	1.09(1.033-1.150)	0.001	56.87%	0.73	0.46
	Finn	MR Egger	20	1.03(0.907-1.178)	0.619			
	Finn	WM	20	1.06(0.988-1.148)	0.097			
	GWAS	IVW	25	0.97(0.888-1.076)	0.191	43.13%		
	GWAS	MR Egger	25	0.97(0.796-1.200)	0.868			
	GWAS	WM	25	0.95(0.838-1.087)	0.527			
TSLP levels	Finn	IVW	22	0.95(0.903-0.999)	0.047	53.92%	0.11	0.90
	Finn	MR Egger	22	0.95(0.846-1.074)	0.446			
	Finn	WM	22	0.94(0.881-1.014)	0.122			
	GWAS	IVW	24	1.07(0.989-1.174)	0.084	46.08%		
	GWAS	MR Egger	24	0.94(0.776-1.141)	0.547			
	GWAS	WM	24	1.13(1.003-1.276)	0.042			

Supplementary Table 3 SNPs associated with eotaxin levels, excluding rs12075

Phenotype	chromosome	Effect allele	Other allele	beta	Standard error	eaf	pval	SNP	samplesize	R2	F
GCST90274764	1	A	G	-0.0767	0.0172	0.1696	8.22E-06	rs10911109	11774	0.001686075	19.88199939
GCST90274764	3	T	C	-0.3316	0.0225	0.9289	3.69E-49	rs2228467	14733	0.014528372	217.1726087
GCST90274764	3	T	C	0.1502	0.0303	0.0433	7.16E-07	rs79684468	14286	0.001717108	24.56935775
GCST90274764	3	T	C	0.0785	0.0123	0.3561	1.75E-10	rs2201150	14286	0.002843033	40.72567455
GCST90274764	3	T	C	0.4043	0.0898	0.0083	6.72E-06	rs148554043	11726	0.001725658	20.26659222
GCST90274764	4	A	C	-0.3374	0.0714	0.0128	2.30E-06	rs139597204	12303	0.001811737	22.32662754
GCST90274764	5	A	C	-0.0656	0.0137	0.2333	1.68E-06	rs6894241	14715	0.001555715	22.92490333
GCST90274764	5	A	G	0.2299	0.0508	0.0222	6.02E-06	rs151240414	11335	0.00180362	20.47735608
GCST90274764	7	A	G	0.2002	0.0205	0.09	1.58E-22	rs757973	14713	0.006440404	95.35893338
GCST90274764	8	A	T	-0.0913	0.0189	0.1104	1.36E-06	rs73209015	14728	0.001581928	23.33237605
GCST90274764	8	T	C	-0.2283	0.0514	0.0172	8.93E-06	rs140965534	14238	0.001383679	19.72534354
GCST90274764	10	T	G	0.1929	0.0422	0.9754	4.85E-06	rs79432525	14247	0.001464467	20.89193099
GCST90274764	11	A	G	-0.0609	0.0131	0.6155	3.34E-06	rs1564995	14286	0.001510514	21.60882686
GCST90274764	11	T	G	0.1017	0.0208	0.9023	1.01E-06	rs34536806	14286	0.001670623	23.90311119
GCST90274764	12	A	G	-0.1138	0.0244	0.9282	3.10E-06	rs35543857	14286	0.001520315	21.74923907
GCST90274764	16	T	C	0.2107	0.0451	0.9774	2.99E-06	rs114744647	14730	0.001479552	21.82312881
GCST90274764	16	T	C	-0.0818	0.0133	0.7358	7.73E-10	rs9940390	14286	0.002640853	37.82182851
GCST90274764	17	T	C	0.0732	0.016	0.8434	4.76E-06	rs8068185	14727	0.001419225	20.92778252
GCST90274764	17	T	C	-0.1215	0.0156	0.167	6.78E-15	rs79722574	14720	0.004104021	60.65189127
GCST90274764	17	A	G	0.1213	0.0272	0.0494	8.21E-06	rs9303380	14286	0.001390173	19.88488068
GCST90274764	17	C	G	-0.1089	0.0244	0.9022	8.08E-06	rs12453306	11335	0.001754252	19.91587864
GCST90274764	18	C	G	0.0555	0.0121	0.5296	4.50E-06	rs55843637	14286	0.001470502	21.03557662
GCST90274764	18	A	G	0.0554	0.0125	0.3264	9.34E-06	rs170520	14285	0.001373164	19.6398739
GCST90274764	19	C	G	0.0817	0.016	0.8362	3.29E-07	rs2112920	14286	0.001821804	26.07013881
GCST90274764	20	T	G	0.2826	0.0606	0.0125	3.11E-06	rs139476527	12933	0.001678689	21.74362257
GCST90274764	21	A	G	-0.1678	0.0345	0.0333	1.15E-06	rs9976282	14722	0.001604285	23.65302656
GCST90274764	22	A	G	-0.0861	0.0187	0.1199	4.14E-06	rs73171545	14286	0.001481728	21.19640874

Supplementary Table 4 Phenotypes associated with 98 metabolites.

Group	ID	Phenotype
Nucleotide	GCST90200407	Uridine levels
	GCST90200413	2'-deoxyuridine levels
Cofactors and vitamins	GCST90200140	Carotene diol (2) levels
Peptide	GCST90200185	Gamma-glutamylcitrulline levels
Amino acid	GCST90200452	Plasma free asparagine levels
	GCST90200303	N-acetyl-L-alanine levels
	GCST90200419	Glutamine levels
	GCST90200305	2-aminobutyrate levels
	GCST90200677	N-acetylphenylalanine levels
	GCST90199915	Imidazole propionate levels
	GCST90199911	Beta-citrylglutamate levels
	GCST90199902	Hydantoin-5-propionate levels
	GCST90199671	Cysteine s-sulfate levels
	GCST90199726	N-acetylthreonine levels
	GCST90199655	4-methyl-2-oxopentanoate levels
	GCST90199971	3-methylglutarylcarnitine (2) levels
	GCST90200432	Threonine levels
	GCST90200192	N,N-dimethylalanine levels
	GCST90200191	N-lactoyl tyrosine levels
	GCST90200404	Ornithine levels
	GCST90200402	Lysine levels
	GCST90200168	Hydroxyasparagine levels
	GCST90200019	Dopamine 3-o-sulfate levels
	GCST90200384	Guanidinoacetate levels
	GCST90200371	3-(4-hydroxyphenyl)lactate levels
Metabolite ratios	GCST90200974	Phosphate to proline ratio
	GCST90200980	Phosphoethanolamine to choline ratio
	GCST90200770	Phenylalanine to tyrosine ratio
	GCST90200768	Phosphate to 2'-deoxyuridine ratio
	GCST90200722	S-adenosylhomocysteine (SAH) to leucine ratio
	GCST90200734	Adenosine 5'-monophosphate (AMP) to palmitate (16:0) ratio
	GCST90200738	Adenosine 5'-monophosphate (AMP) to inosine 5'-monophosphate (IMP) ratio
	GCST90200744	Arginine to glutamate ratio
	GCST90200749	Arachidonate (20:4n6) to pyruvate ratio
	GCST90200758	Glycine to serine ratio
	GCST90200893	Taurine to cysteine ratio
	GCST90200902	hosphate to linoleoyl-arachidonoyl-glycerol (18:2 to 20:4) [2] ratio
	GCST90200906	2'-deoxyuridine to cytidine ratio
	GCST90200947	Adenosine 5'-diphosphate (ADP) to glucose ratio
	GCST90200957	N-acetylneuraminate to N-acetylglucosamine to N-acetylgalactosamine ratio
	GCST90200960	Choline phosphate to phosphoethanolamine ratio
	GCST90200921	Mannose to mannitol to sorbitol ratio

	GCST90200883	Phosphate to acetoacetate ratio
	GCST90200888	Citrate to 4-hydroxyphenylpyruvate ratio
	GCST90200842	Citrate to oxalate (ethanedioate) ratio
	GCST90200806	Spermidine to ergothioneine ratio
	GCST90200832	Adenosine 5'-diphosphate (ADP) to citrate ratio
	GCST90199787	Homostachydrine levels
	GCST90199632	Tartarate levels
	GCST90199690	EDTA levels
	GCST90199998	Propyl 4-hydroxybenzoate sulfate levels
Xenobiotics	GCST90199857	5alpha-pregnan-diol disulfate levels
	GCST90199869	2-hydroxyhippurate levels
	GCST90201017	4-methyl-2-oxopentanoate to 3-methyl-2-oxobutyrate ratio
	GCST90200166	Dihydroferulic acid sulfate levels
	GCST90200491	X-12026 levels
	GCST90200464	X-11849 levels
	GCST90200461	X-10458 levels
	GCST90200512	X-13684 levels
	GCST90200502	X-12707 levels
	GCST90200538	X-17010 levels
Unknow	GCST90200710	X-13431 levels
	GCST90200553	X-17653 levels
	GCST90200646	X-24801 levels
	GCST90200640	X-24978 levels
	GCST90200617	X-23665 levels
	GCST90200618	X-23782 levels
	GCST90200172	3-carboxy-4-methyl-5-pentyl-2-furanpropionate (3-CMPFP) levels
	GCST90200109	Linoleoyl-arachidonoyl-glycerol (18:2/20:4) [2] levels
	GCST90200111	N-stearoyl-sphingadienine (d18:2/18:0) levels
	GCST90200332	1-palmitoyl-2-linoleoyl-GPI (16:0/18:2) levels
	GCST90200260	Branched chain 14:0 dicarboxylic acid levels
	GCST90199845	Sphinganine-1-phosphate levels
	GCST90199852	5alpha-androstan-3beta,17alpha-diol disulfate levels
	GCST90200088	(S)-3-hydroxybutyrylcarnitine levels
	GCST90200067	1-stearoyl-2-linoleoyl-GPI (18:0/18:2) levels
Lipid	GCST90199968	1-palmitoyl-GPG (16:0) levels
	GCST90199970	Octadecenedioylcarnitine (C18:1-DC) levels
	GCST90200018	Adipoylcarnitine (C6-DC) levels
	GCST90199992	Octadecanedioylcarnitine (C18-DC) levels
	GCST90199997	Behenoyl sphingomyelin (d18:1/22:0) levels
	GCST90199712	3-carboxy-4-methyl-5-propyl-2-furanpropanoate (cmpf) levels
	GCST90199718	Sebacate (C10-DC) levels
	GCST90199731	10-heptadecenoate (17:1n7) levels
	GCST90199684	1-linoleoylglycerol (18:2) levels
	GCST90199899	1-(1-enyl-palmitoyl)-GPC (p-16:0) levels

	GCST90199913	Docosadioate (C22-DC) levels
	GCST90200036	1-palmitoleoylglycerol (16:1) levels
	GCST90200048	1-(1-enyl-palmitoyl)-2-arachidonoyl-GPE (p-16:0/20:4) levels
	GCST90200052	1-(1-enyl-palmitoyl)-2-arachidonoyl-gpc (p-16:0/20:4) levels
	GCST90199795	2-hydroxypalmitate levels
	GCST90199805	Octadecanedioate levels
	GCST90199770	1-arachidonylglycerol (20:4) levels
	GCST90199733	1-palmitoyl-GPC (16:0) levels
	GCST90199734	Hyocholate levels
	GCST90199750	Laurylcarnitine levels
	GCST90200238	Pregnenetriol disulfate levels
	GCST90199792	1-palmitoyl-GPE (16:0) levels

1. Eotaxin levels-forest

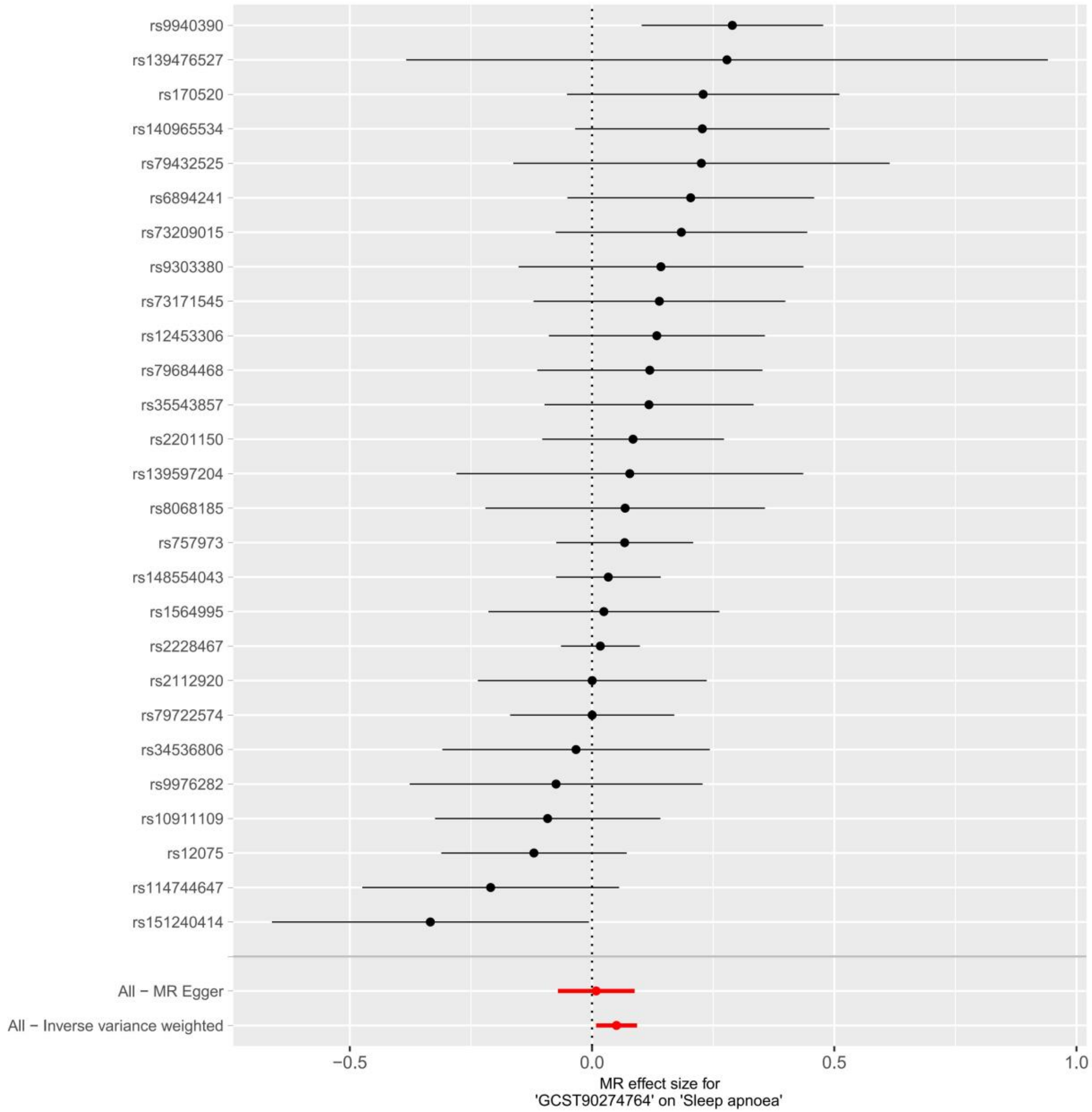

2.Eotaxin levels-scatter

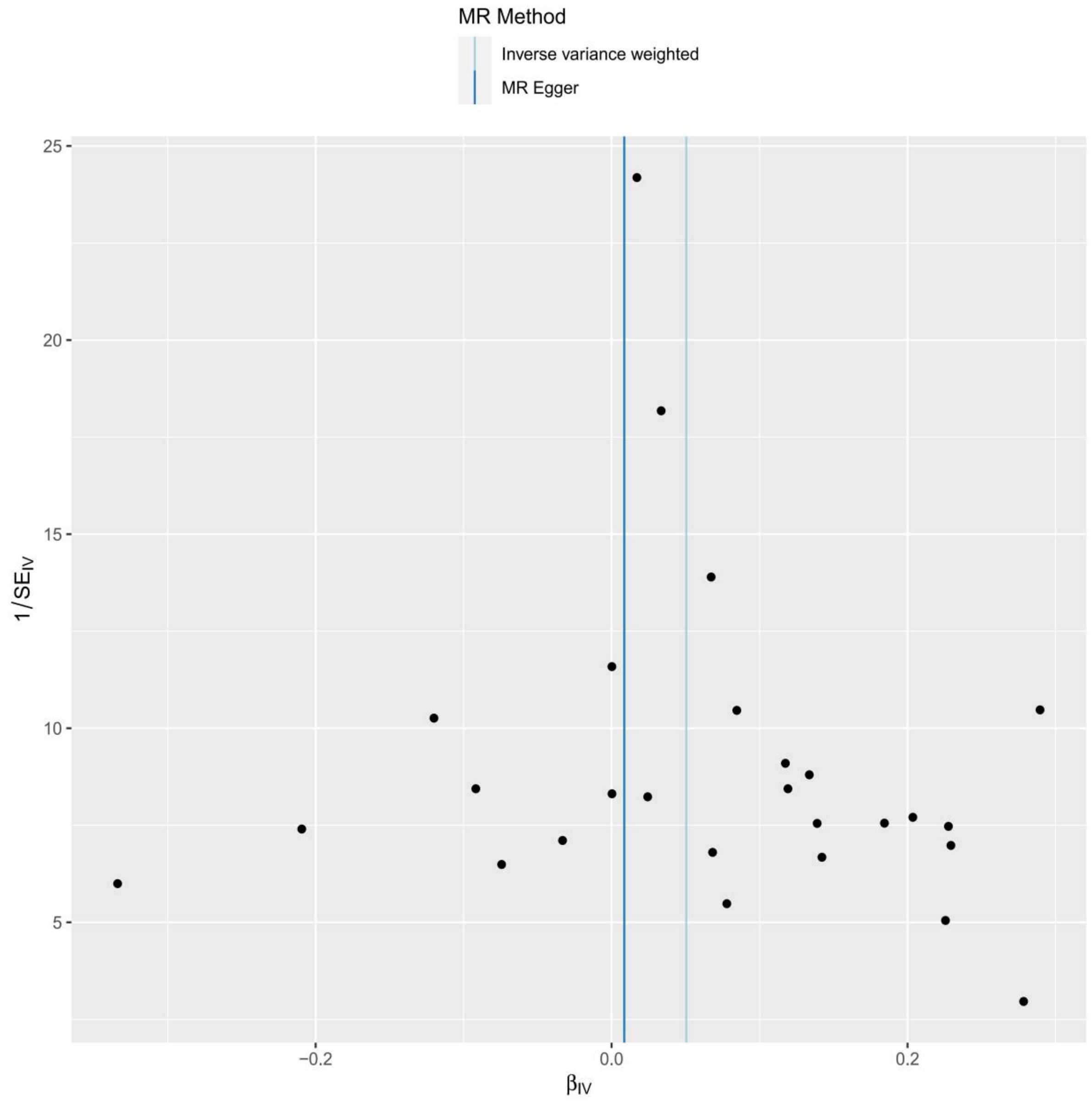

3.Eotaxin levels-funnelplot

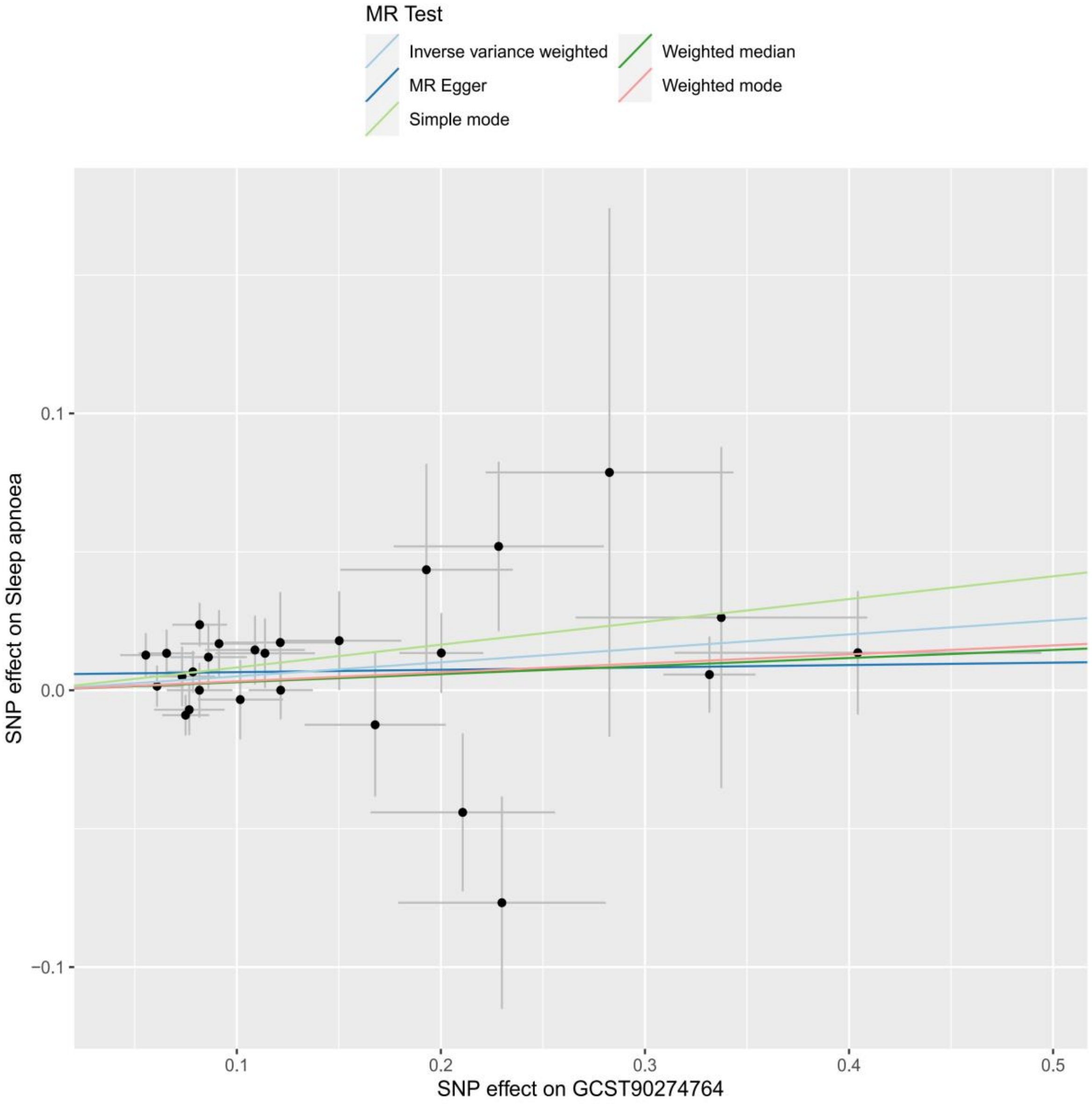

4. Eotaxin levels-leave one out

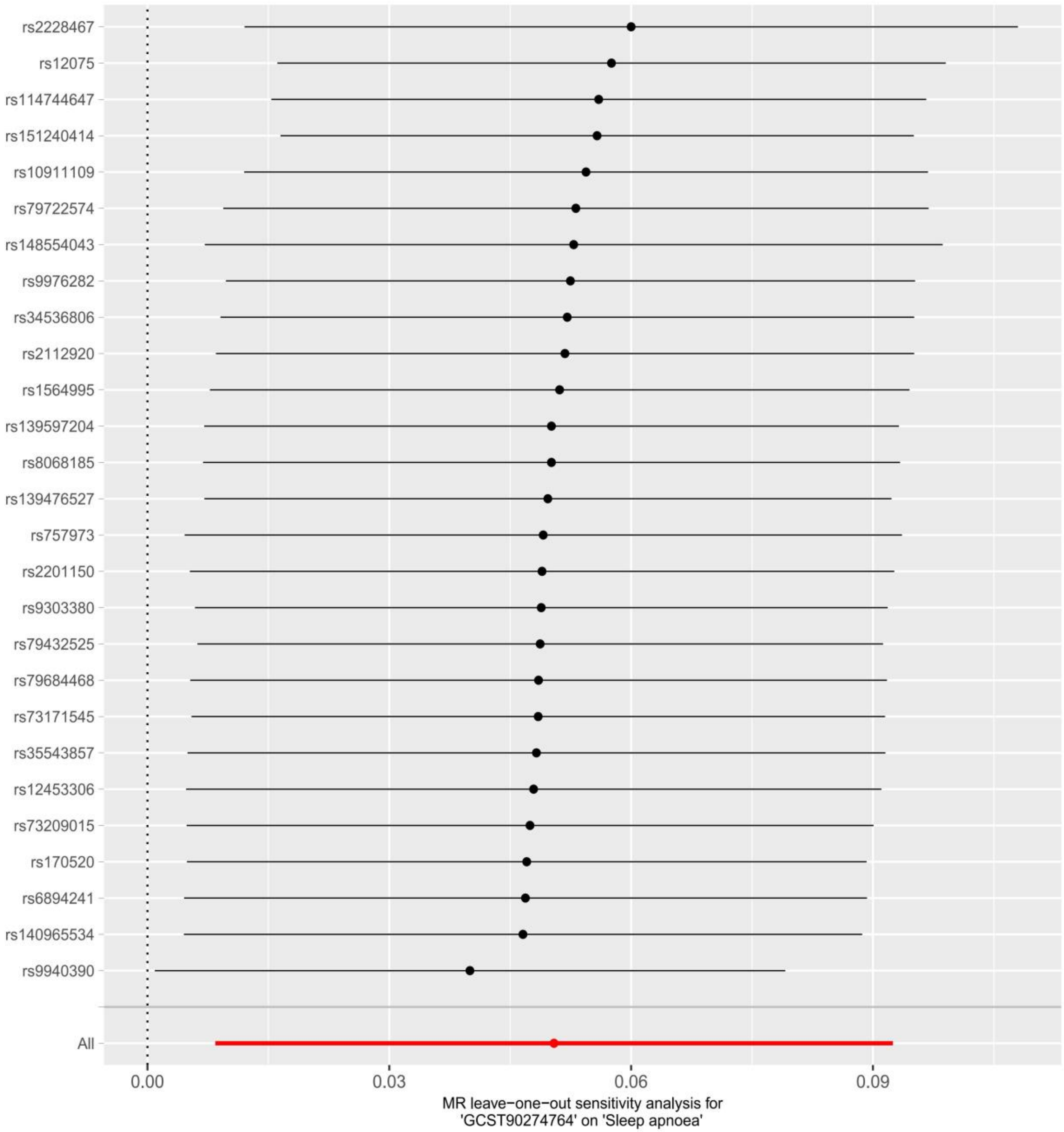

5. T-cell surface glycoprotein CD6 isoform levels-forest

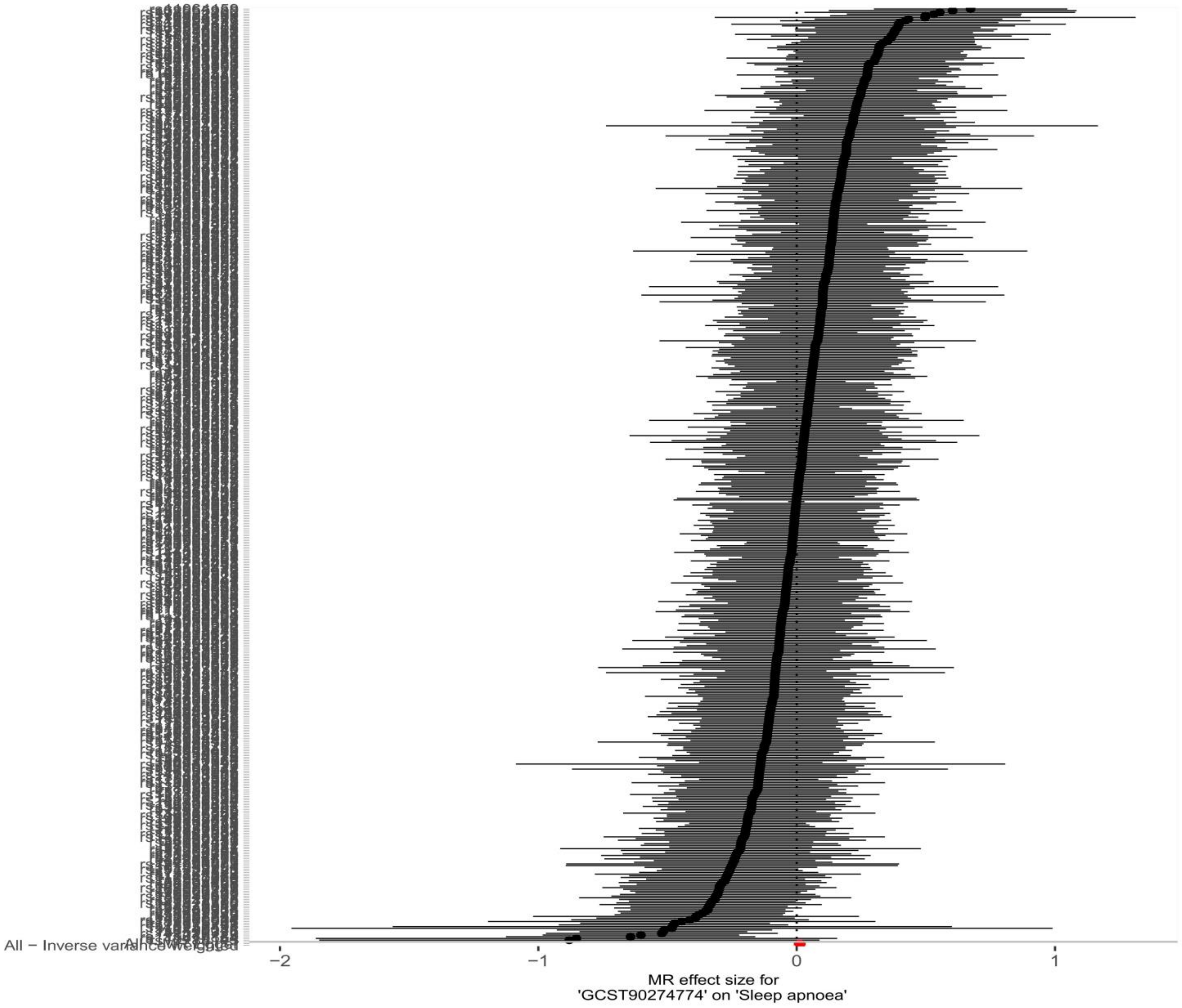

6. T-cell surface glycoprotein CD6 isoform levels-funnelplot

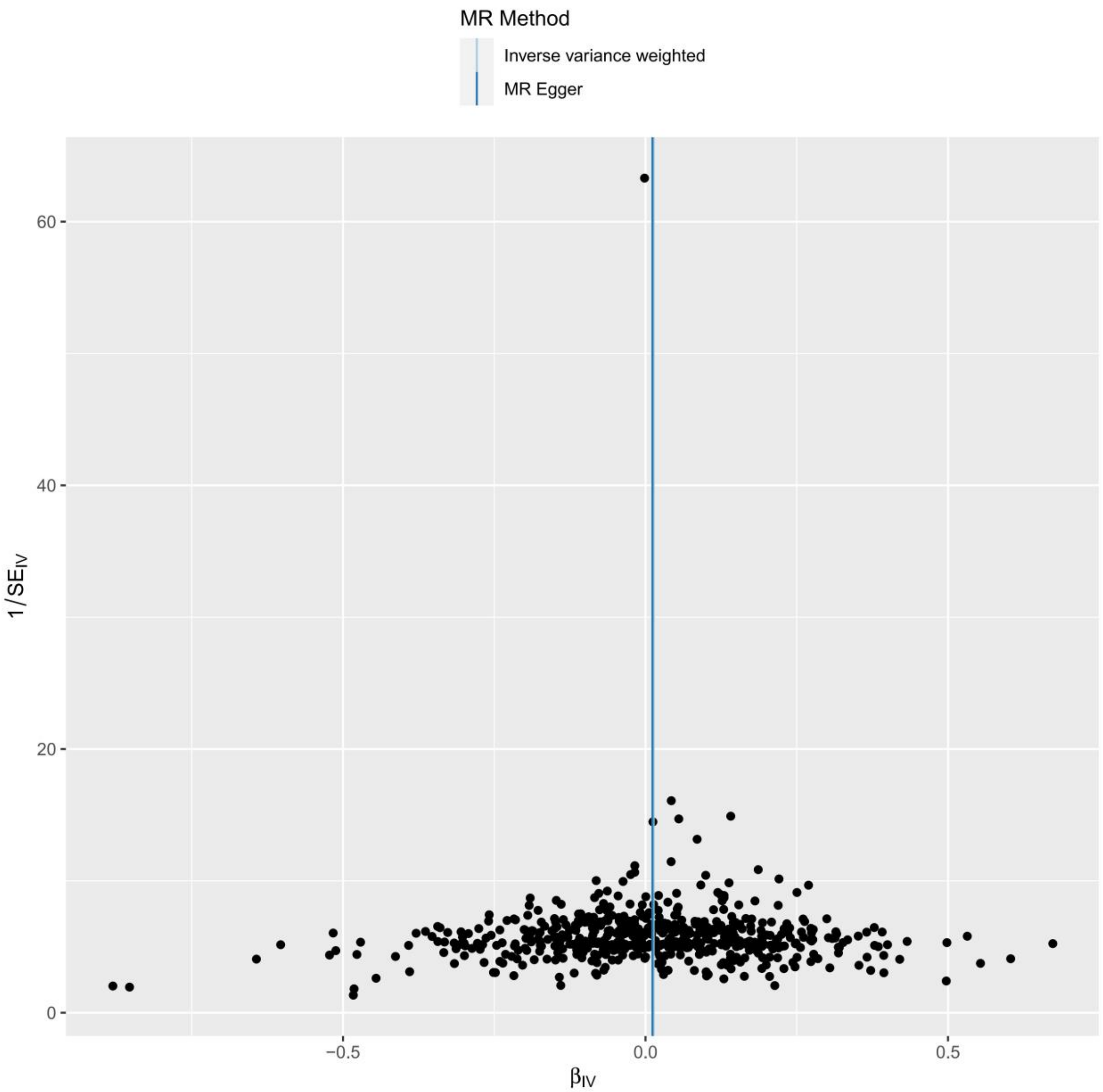

7. T-cell surface glycoprotein CD6 isoform levels-scatter

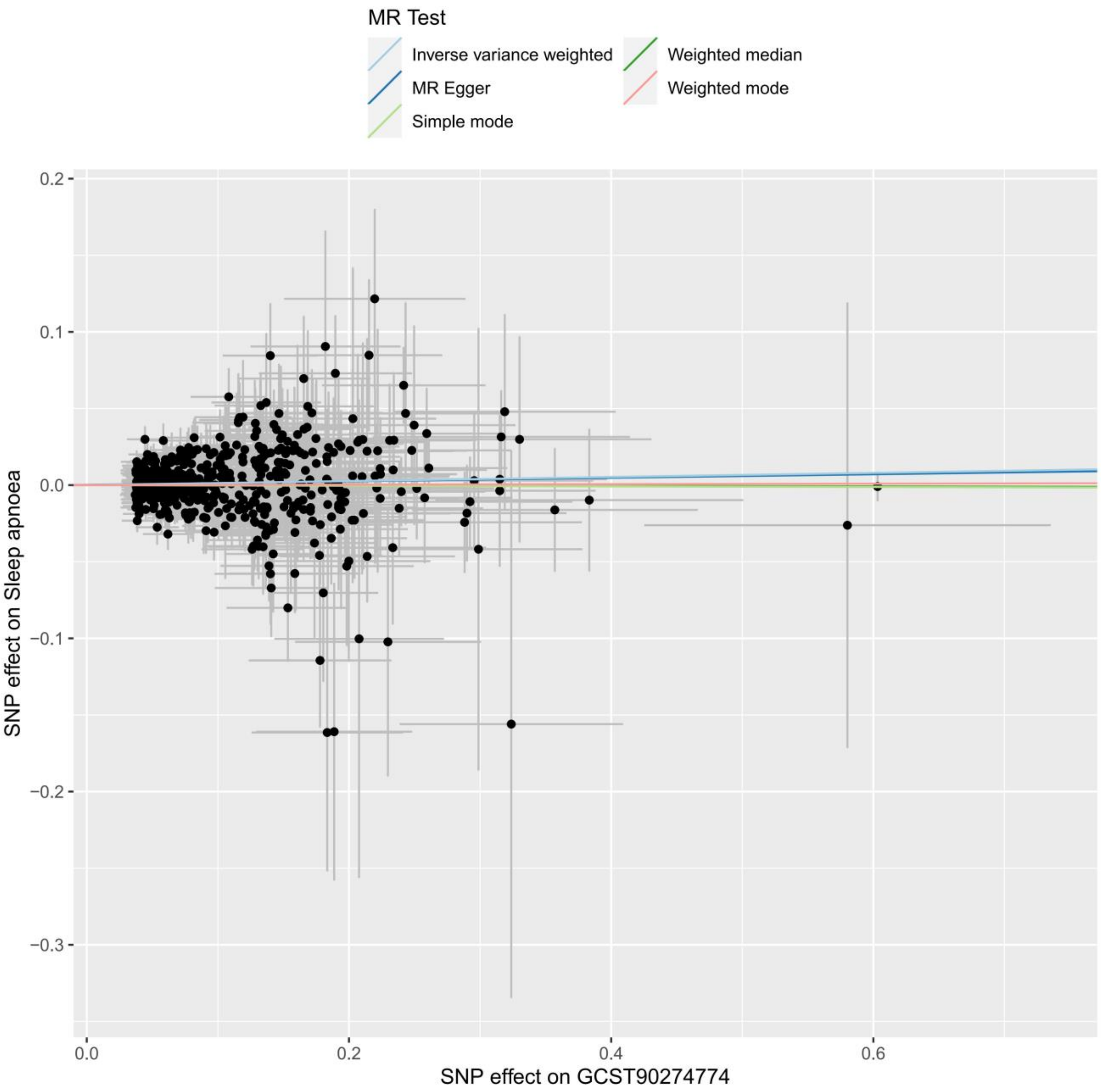

8. T-cell surface glycoprotein CD6 isoform levels -leave one out

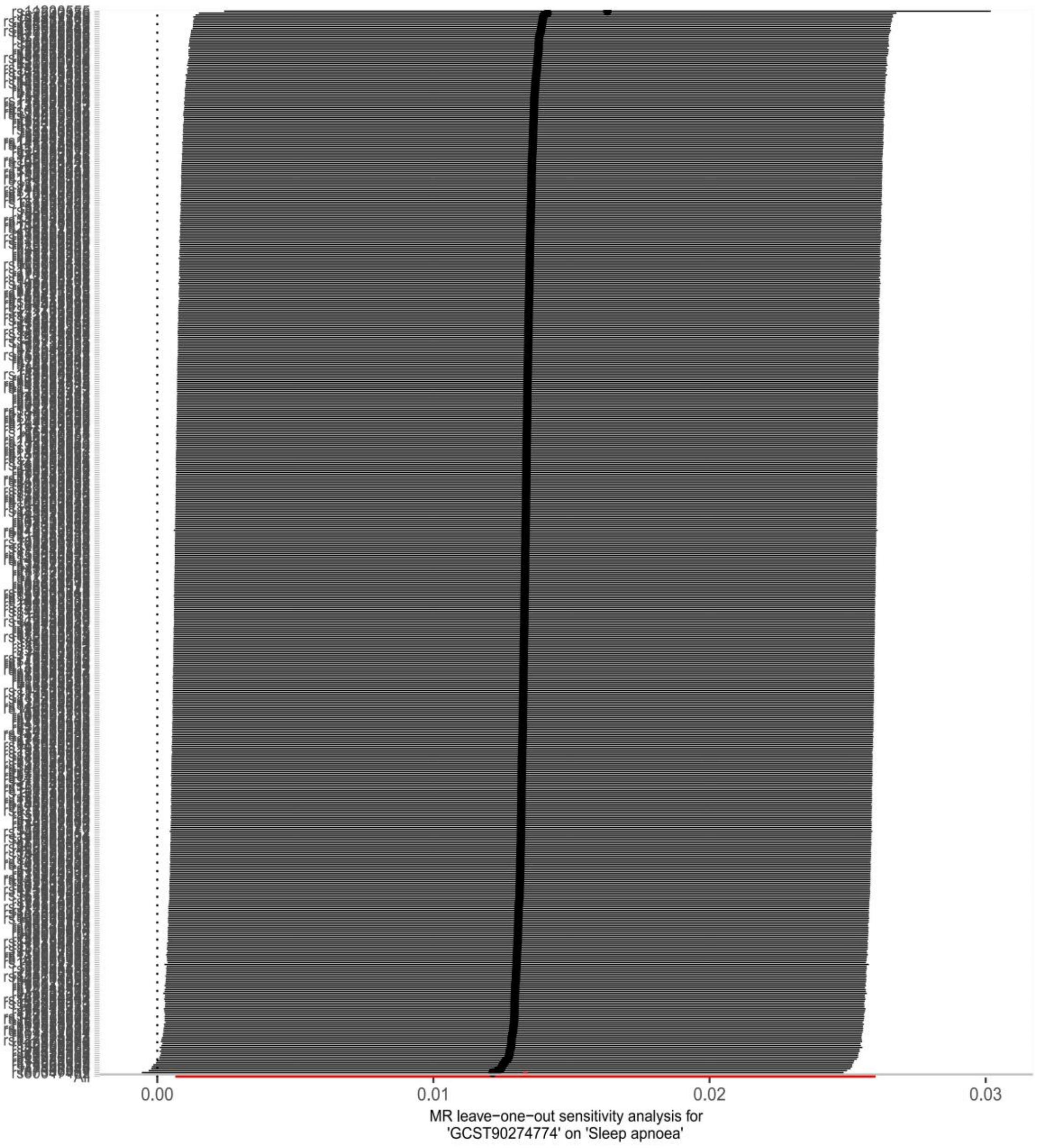

9. Glial cell line-derived neurotrophic factor levels-forest

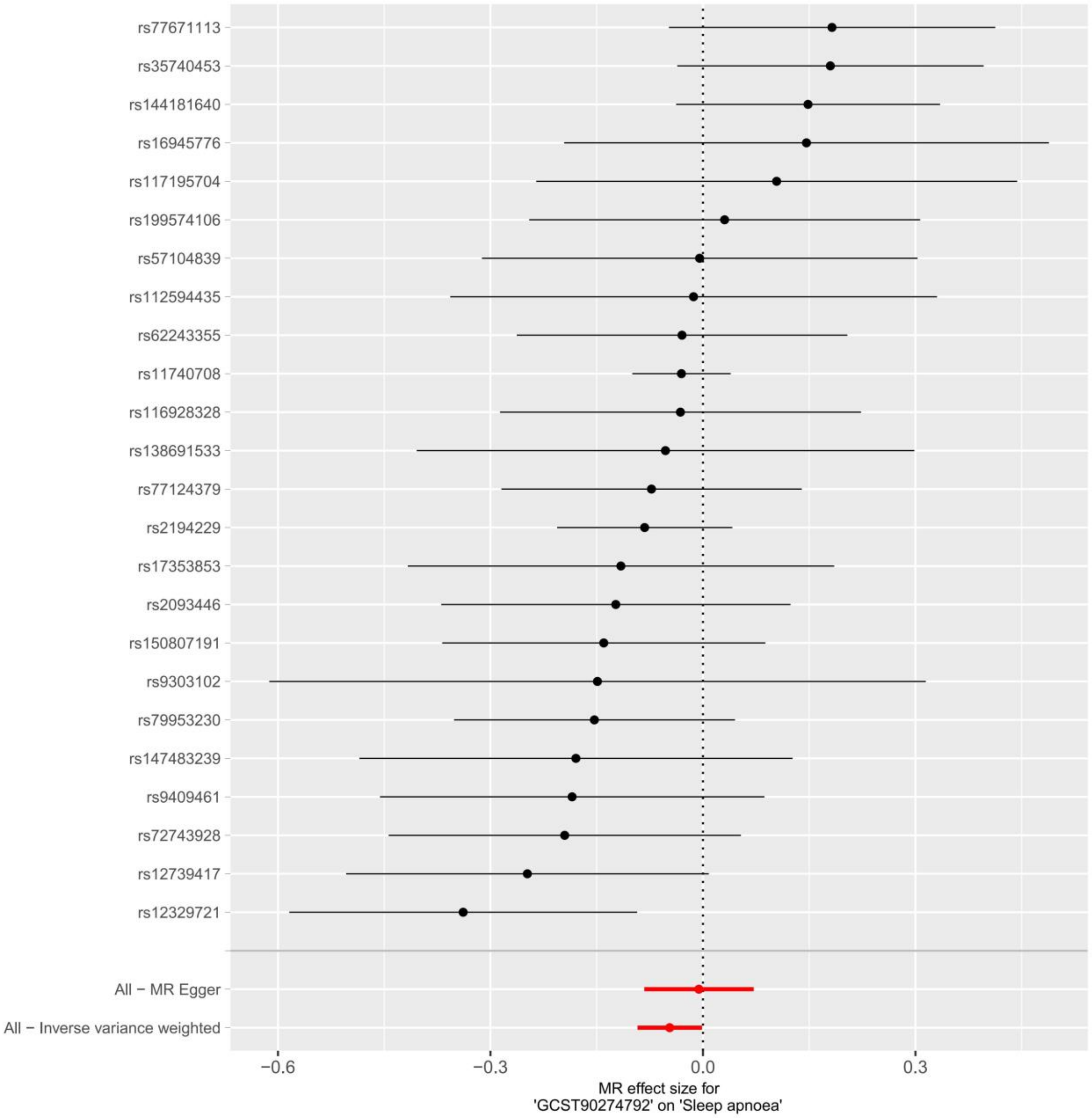

10.Glial cell line-derived neurotrophic factor levels-funnelplot

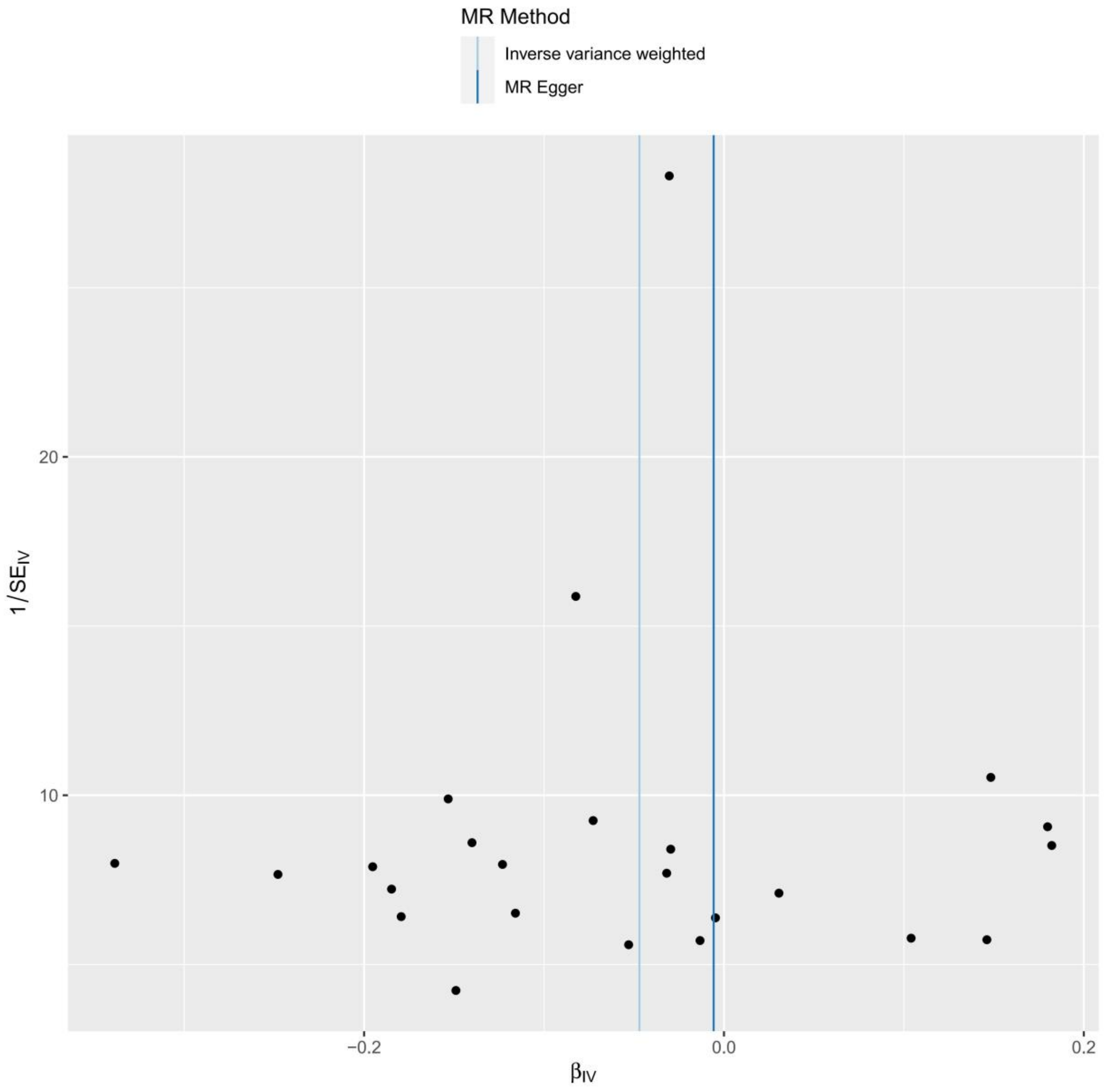

11. Glial cell line-derived neurotrophic factor levels-scatter

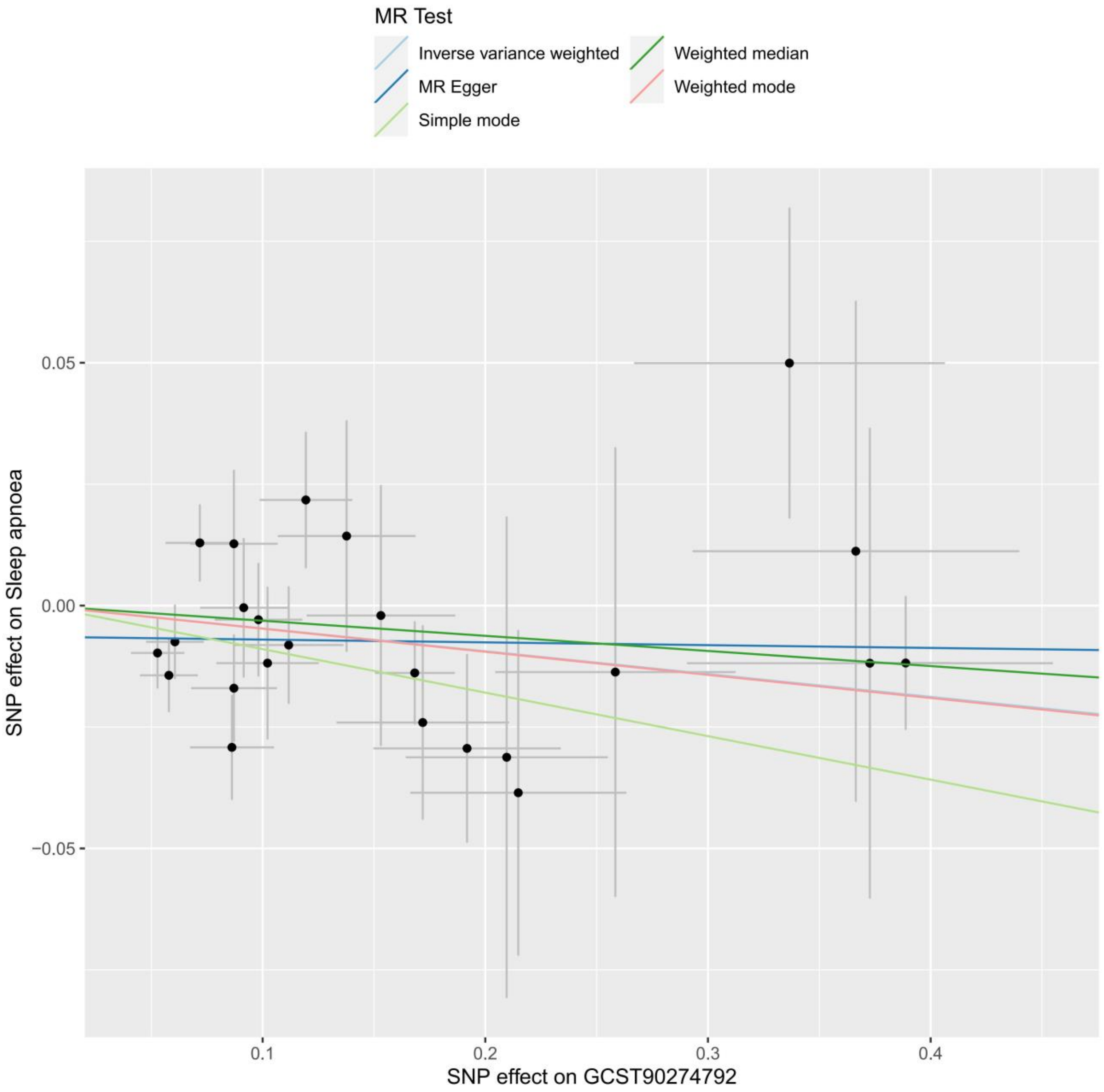

12. Glial cell line-derived neurotrophic factor levels-leave one out

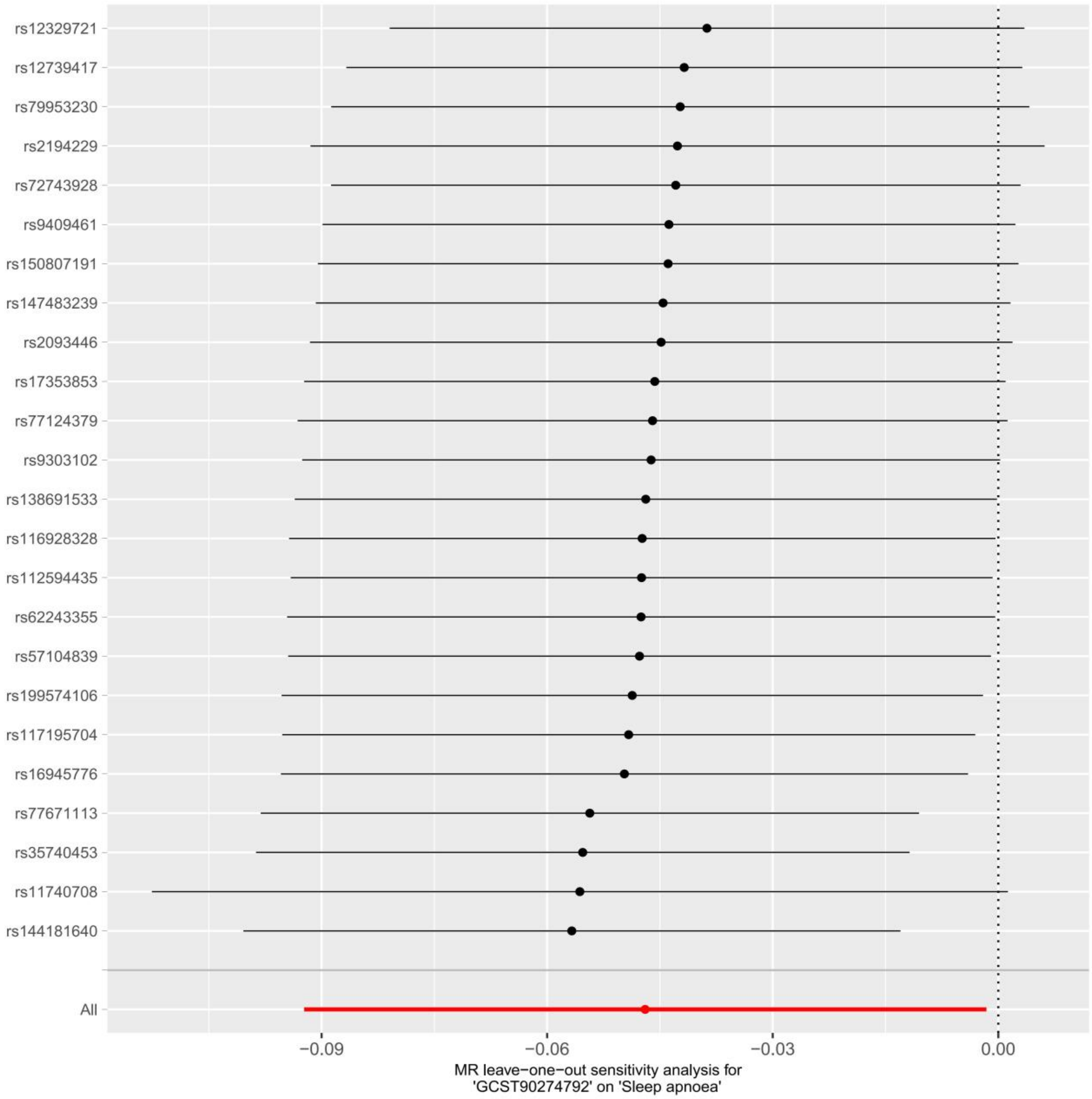

13. Interleukin-20 levels -forest

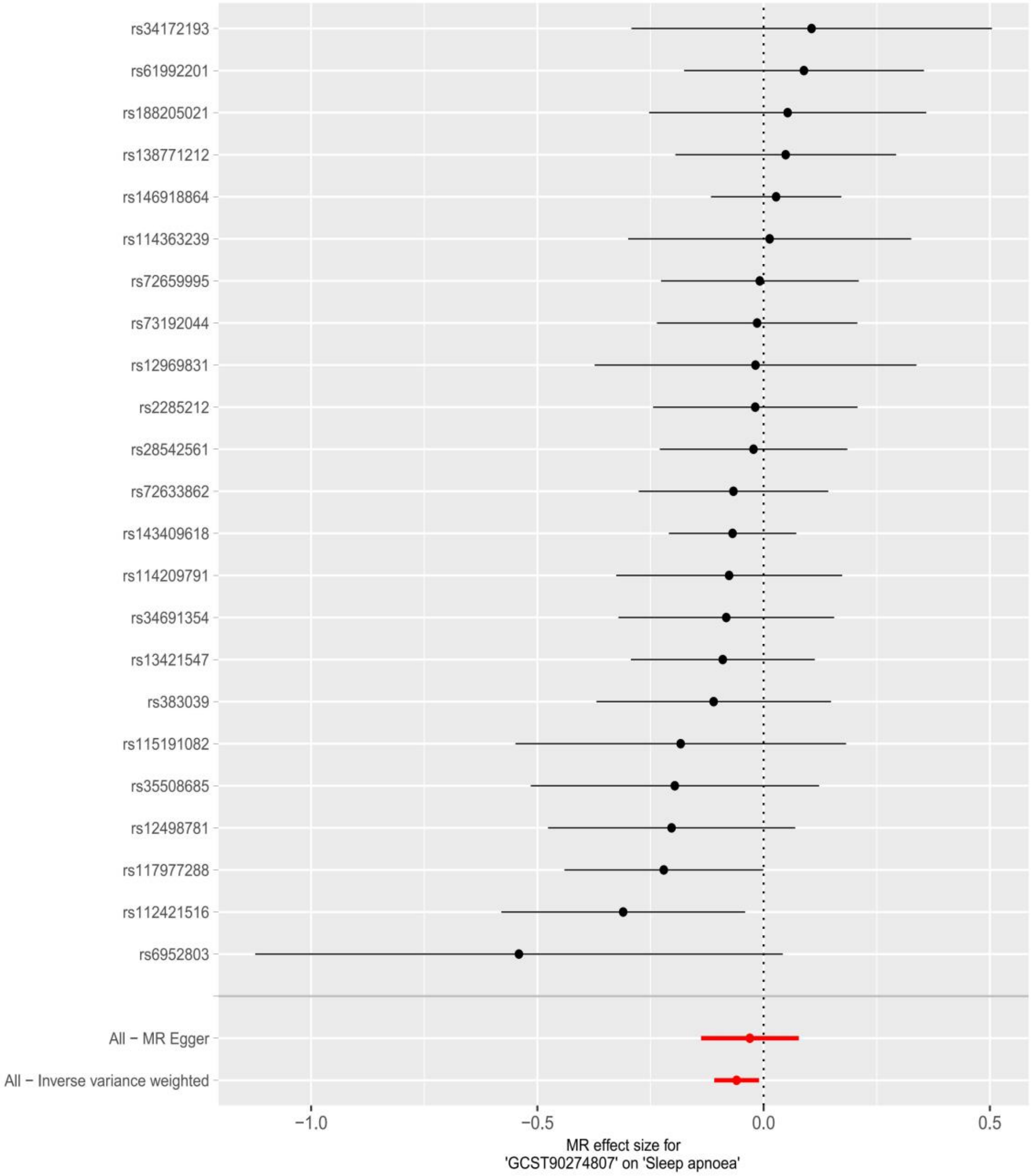

14. Interleukin-20 levels-funnelplot

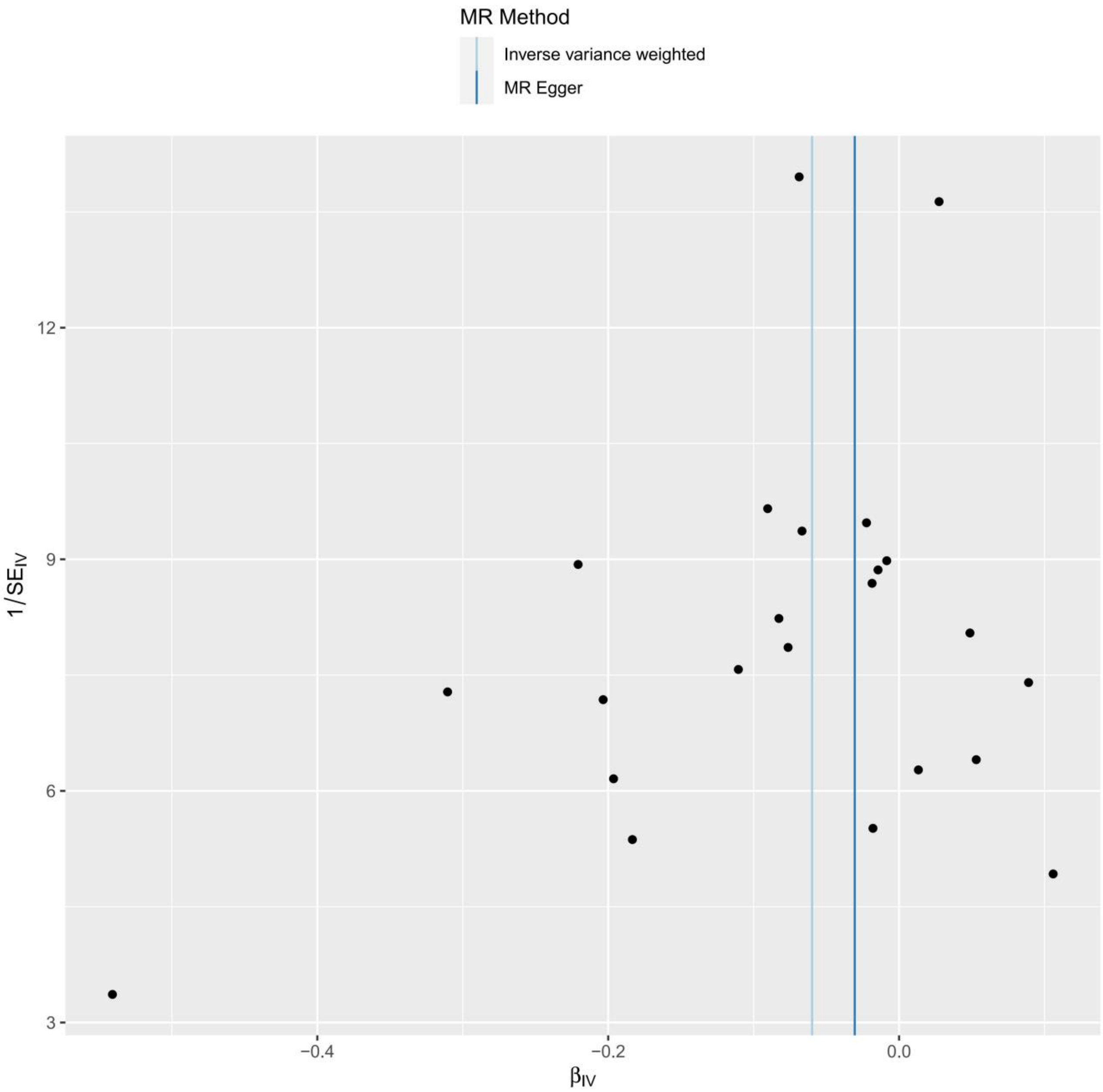

15. Interleukin-20 levels -scatter

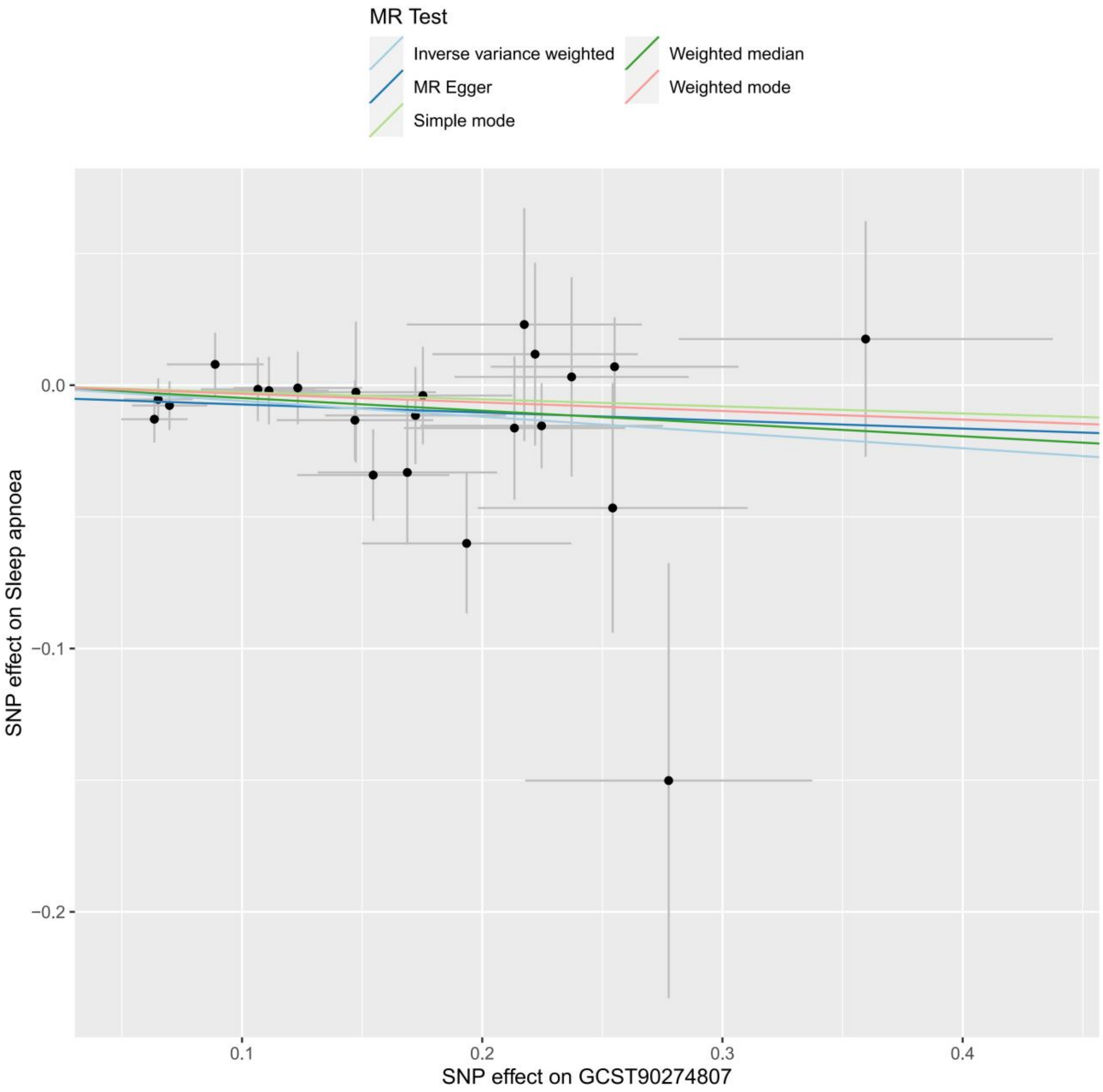

16. Interleukin-20 levels -leave one out

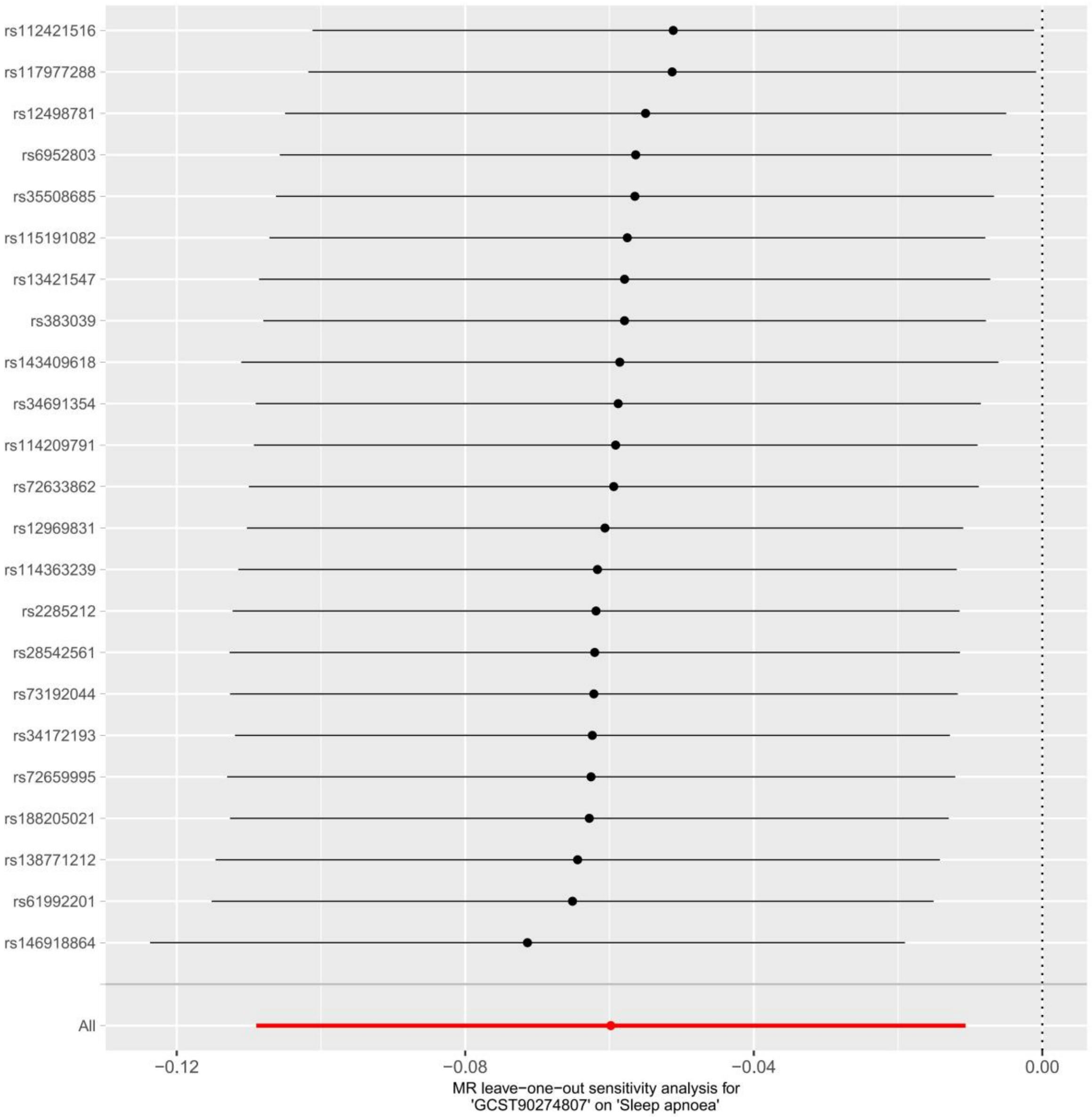

17. Interleukin-20 receptor subunit alpha levels-forest

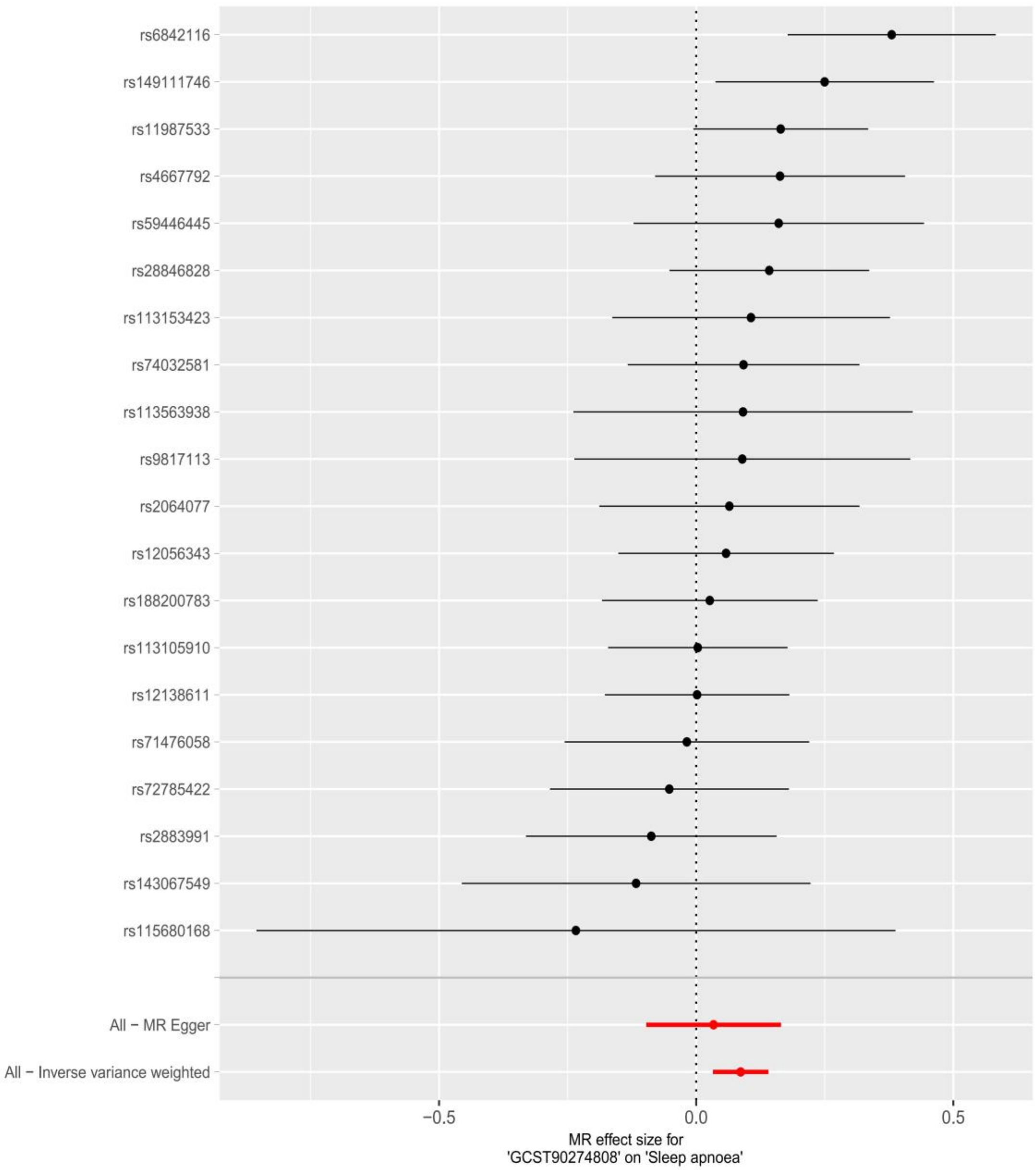

18. Interleukin-20 receptor subunit alpha levels -funnelplot

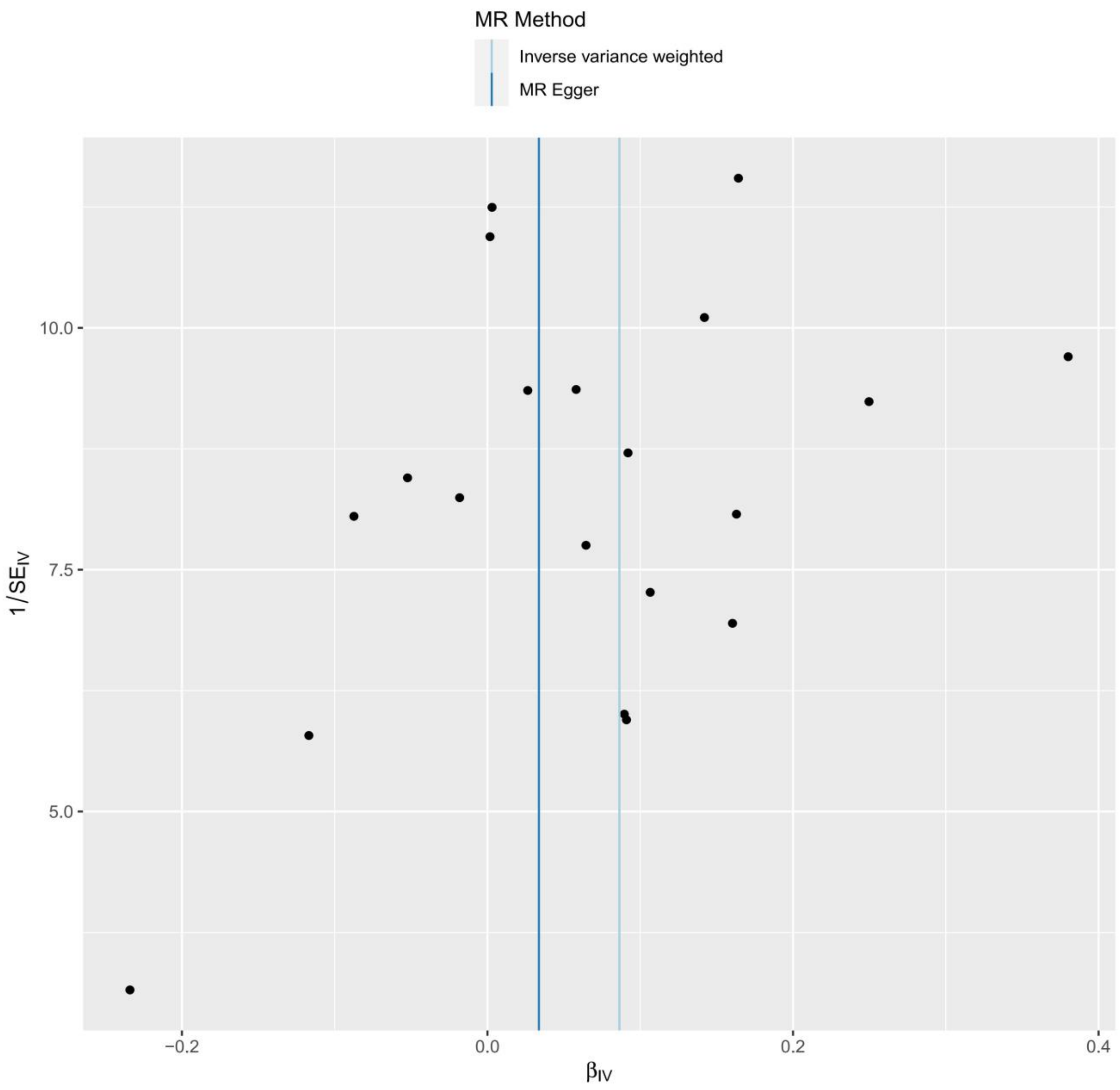

19. Interleukin-20 receptor subunit alpha levels-scatter

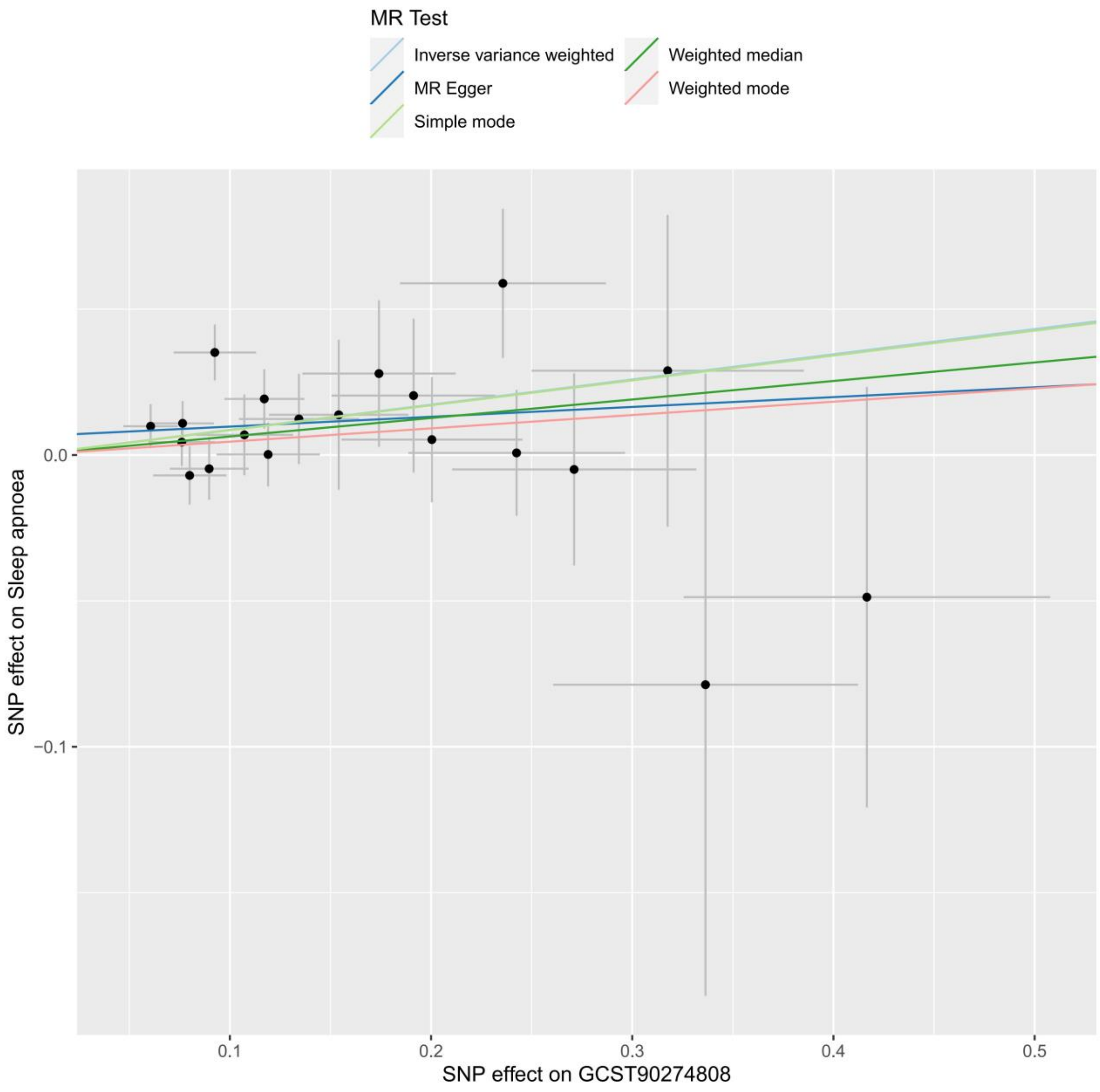

20. Interleukin-20 receptor subunit alpha levels-leave one out

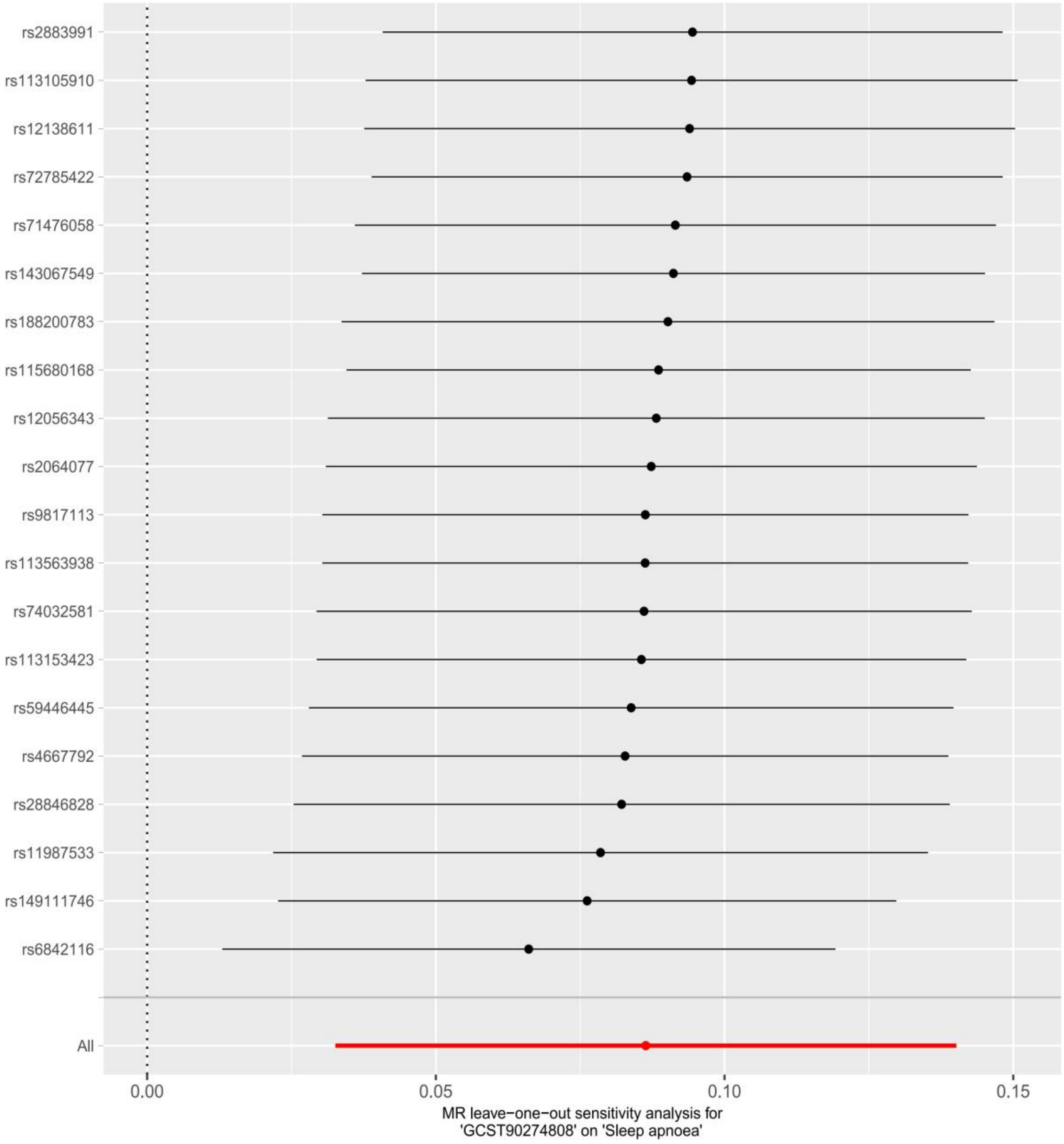

21. Thymic stromal lymphopoietin levels -forest

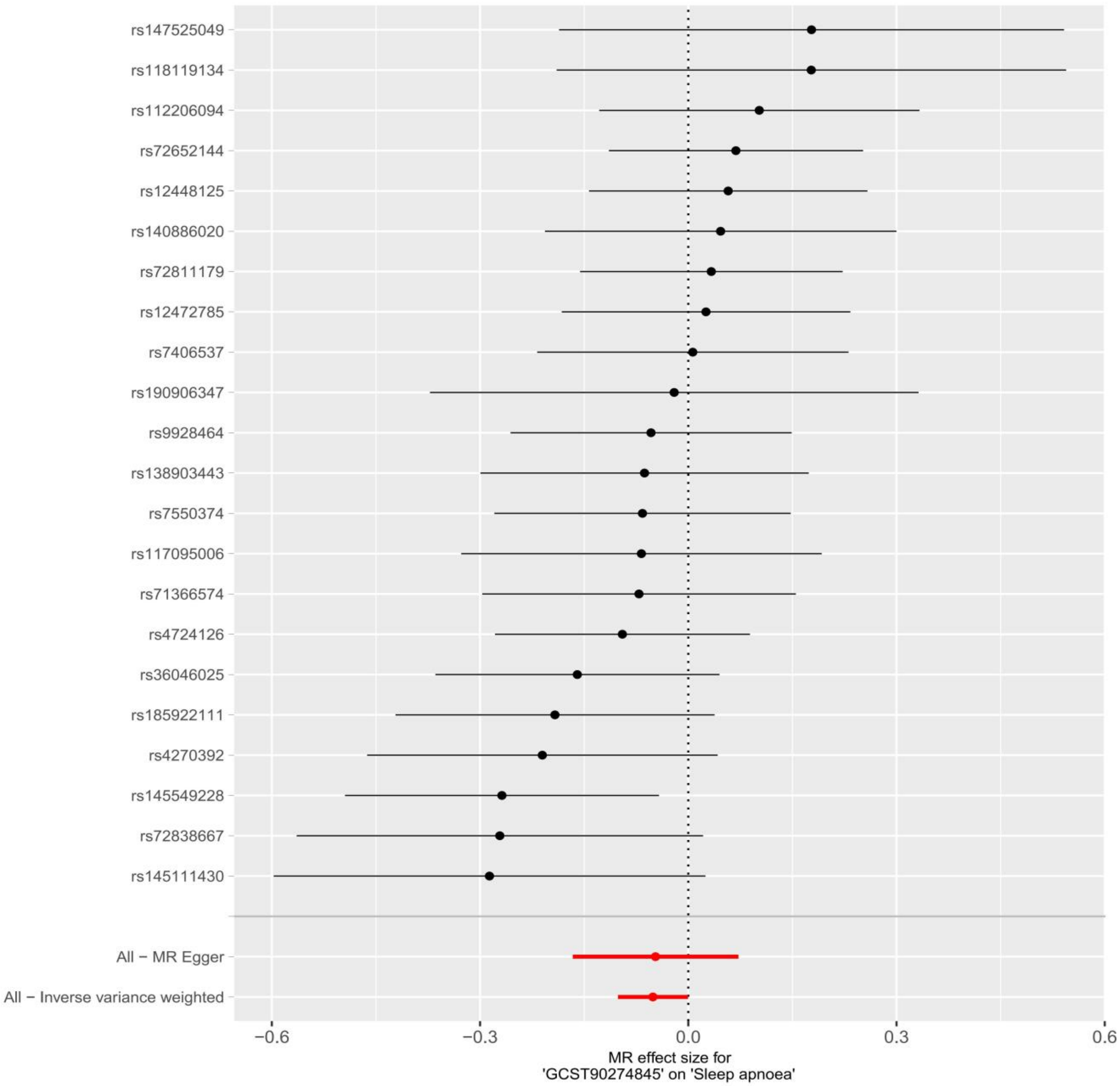

22. Thymic stromal lymphopoietin levels -funnelplot

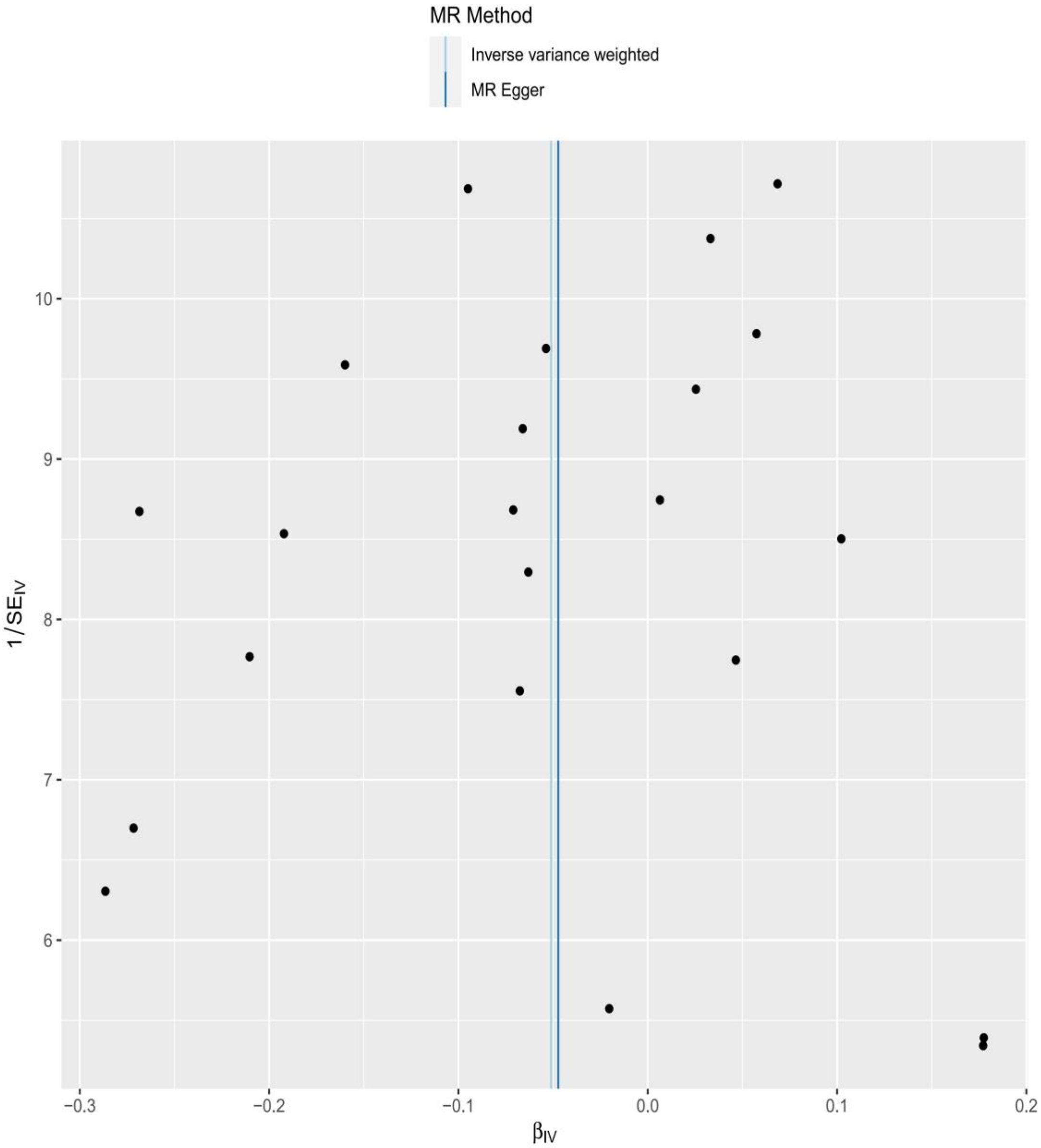

23. Thymic stromal lymphopoietin levels -scatter

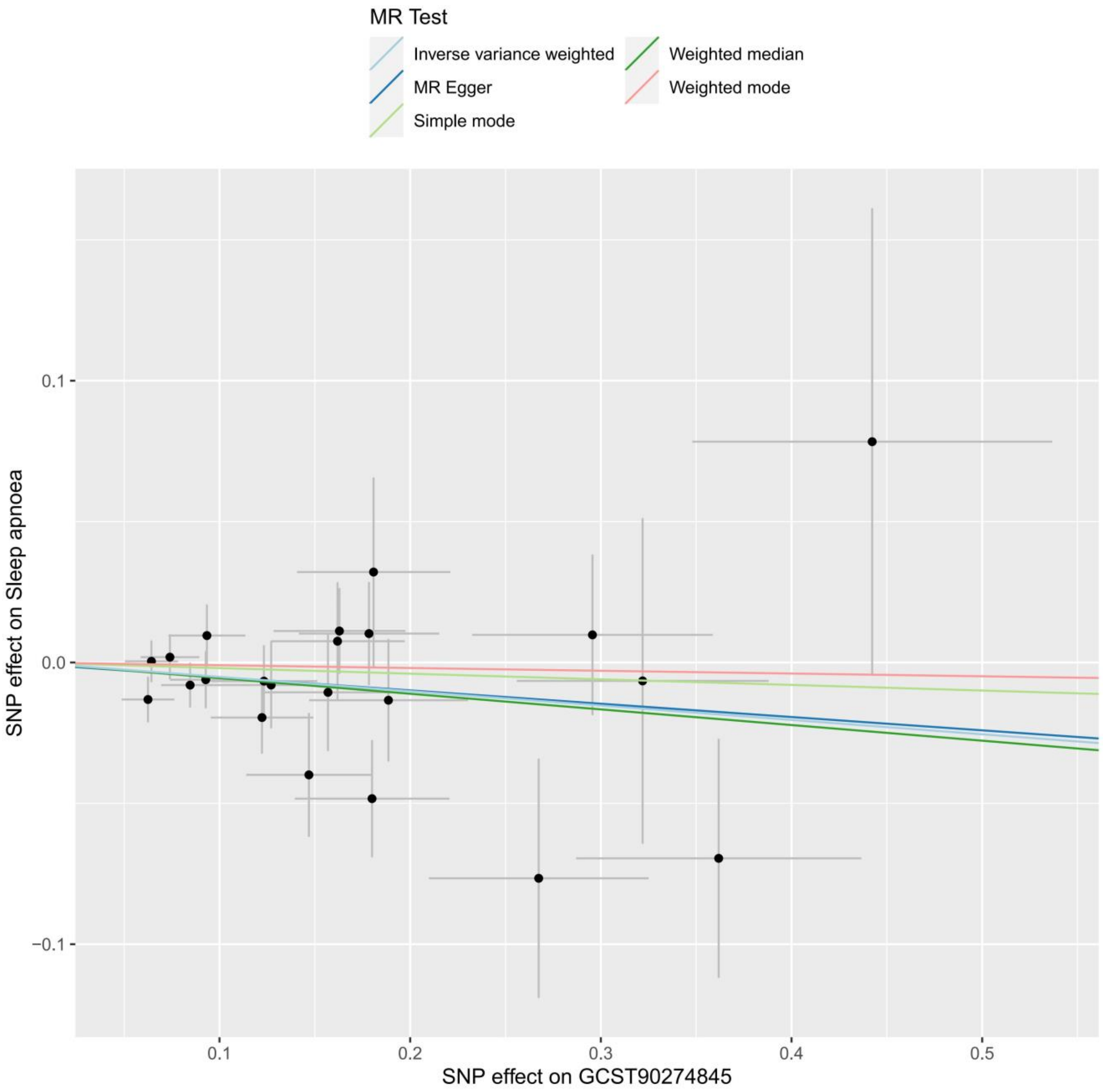

24. Thymic stromal lymphopoietin levels -leave one out

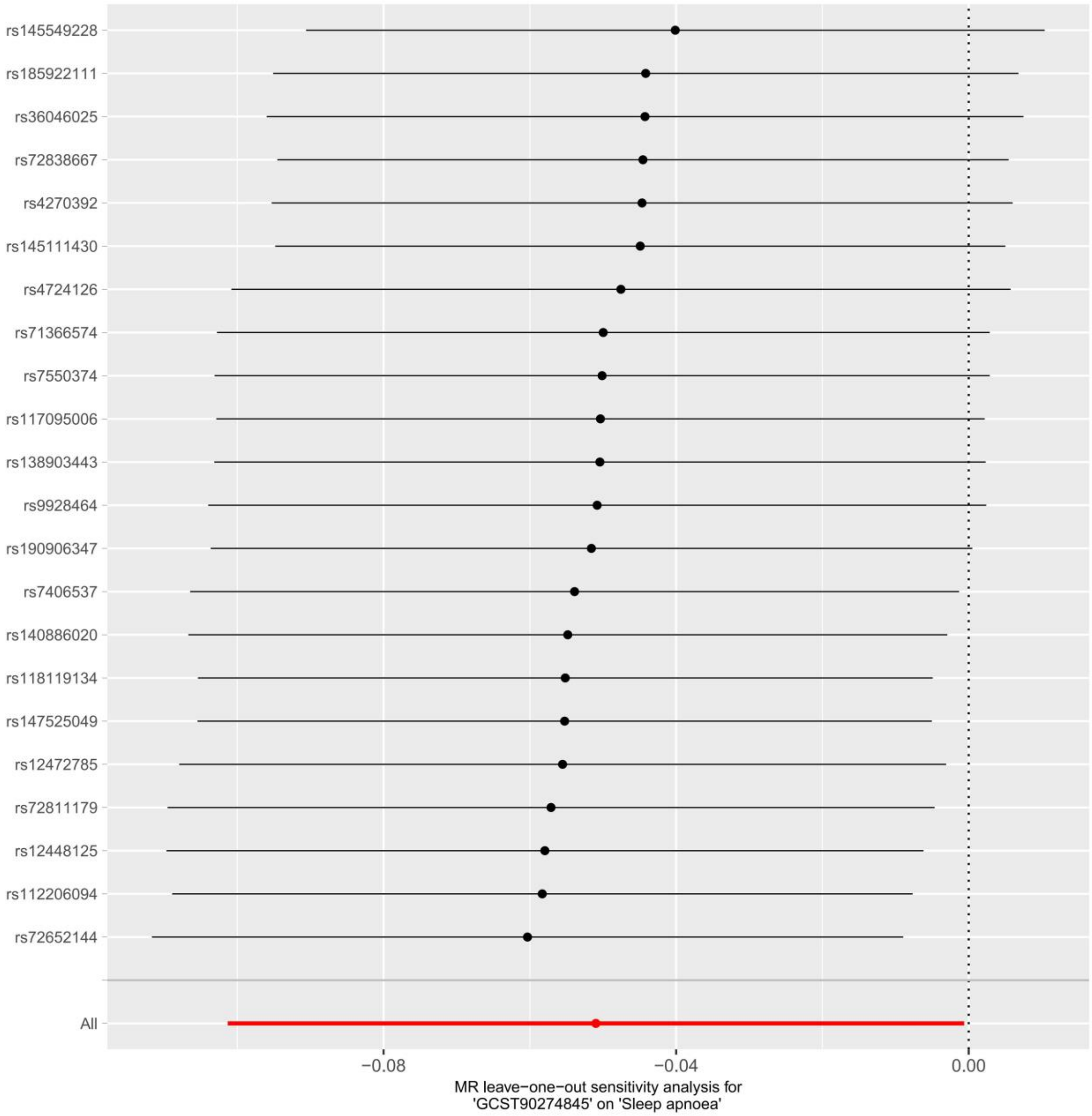

Supplement: Supplementary file 1 [file Table1.pdf]
